# Multi-omics data integration reveals link between epigenetic modifications and gene expression in sugar beet (*Beta vulgaris subsp. vulgaris*) in response to cold

**DOI:** 10.1186/s12864-022-08312-2

**Published:** 2022-02-17

**Authors:** Sindy Gutschker, José María Corral, Alfred Schmiedl, Frank Ludewig, Wolfgang Koch, Karin Fiedler-Wiechers, Olaf Czarnecki, Karsten Harms, Isabel Keller, Cristina Martins Rodrigues, Benjamin Pommerrenig, H. Ekkehard Neuhaus, Wolfgang Zierer, Uwe Sonnewald, Christina Müdsam

**Affiliations:** 1 Biochemistry, Friedrich-Alexander University Erlangen-Nürnberg (FAU), Staudtstraße 5, 91058 Erlangen, Germany; 2 KWS SAAT SE & Co. KGaA, Grimsehlstraße 31, 37574 Einbeck, Germany; 3 Südzucker AG, Central Department for Research, Development, and Service, Wormser Straße 11, 67283 Obrigheim/Pfalz, Germany; 4 Plant Physiology, TU Kaiserslautern, Building 22, Erwin-Schrödinger-Straße, 67663 Kaiserslautern, Germany

**Keywords:** *Beta vulgaris subsp. vulgaris*, Abiotic stress, Cold response, WGBS, RNA-seq, DNA methylation, Epigenetics, Omics

## Abstract

**Background:**

DNA methylation is thought to influence the expression of genes, especially in response to changing environmental conditions and developmental changes. Sugar beet (*Beta vulgaris ssp. vulgaris*), and other biennial or perennial plants are inevitably exposed to fluctuating temperatures throughout their lifecycle and might even require such stimulus to acquire floral competence. Therefore, plants such as beets, need to fine-tune their epigenetic makeup to ensure phenotypic plasticity towards changing environmental conditions while at the same time steering essential developmental processes. Different crop species may show opposing reactions towards the same abiotic stress, or, vice versa, identical species may respond differently depending on the specific kind of stress.

**Results:**

In this study, we investigated common effects of cold treatment on genome-wide DNA methylation and gene expression of two *Beta vulgaris* accessions via multi-omics data analysis. Cold exposure resulted in a pronounced reduction of DNA methylation levels, which particularly affected methylation in CHH context (and to a lesser extent CHG) and was accompanied by transcriptional downregulation of the chromomethyltransferase *CMT2* and strong upregulation of several genes mediating active DNA demethylation.

**Conclusion:**

Integration of methylomic and transcriptomic data revealed that, rather than methylation having directly influenced expression, epigenetic modifications correlated with changes in expression of known players involved in DNA (de)methylation. In particular, cold triggered upregulation of genes putatively contributing to DNA demethylation via the ROS1 pathway. Our observations suggest that these transcriptional responses precede the cold-induced global DNA-hypomethylation in non-CpG, preparing beets for additional transcriptional alterations necessary for adapting to upcoming environmental changes.

**Supplementary Information:**

The online version contains supplementary material available at 10.1186/s12864-022-08312-2.

## Background

Sugar beet is the main source of sugar production in Europe. Its fleshy taproot provides around 15% of the world’s annual production of sugar, solely competing with sugar cane in industrial sucrose extraction worldwide [1]. Conventionally, sugar beets are sown in spring and harvested before winter, still in the vegetative stage. In contrast, sowing sugar beet in autumn and harvesting in the following year could increase - due to the development in the winter and the acceleration of growth in the spring - the sugar content by up to 26% [2]. Therefore, one of the major goals in sugar beet breeding is the production of winter beets with the challenge to ensure the viability over the winter season, since particularly seasonal environmental stresses such as frost limit possible periods for their cultivation and thereby also restrict potential enhancement of yield. Biennial sugar beets grow vegetatively in the first season and after extended exposure to cold during the winter season, they switch to generative reproduction and acquire floral competence, as a result of vernalization and the subsequent shift towards long-day conditions in spring [3, 4]. The shoot outgrowth or stem elongation, so-called bolting, and the following flower development drastically reduce the sugar yield and the size of the beet.

**Fig. 1:**
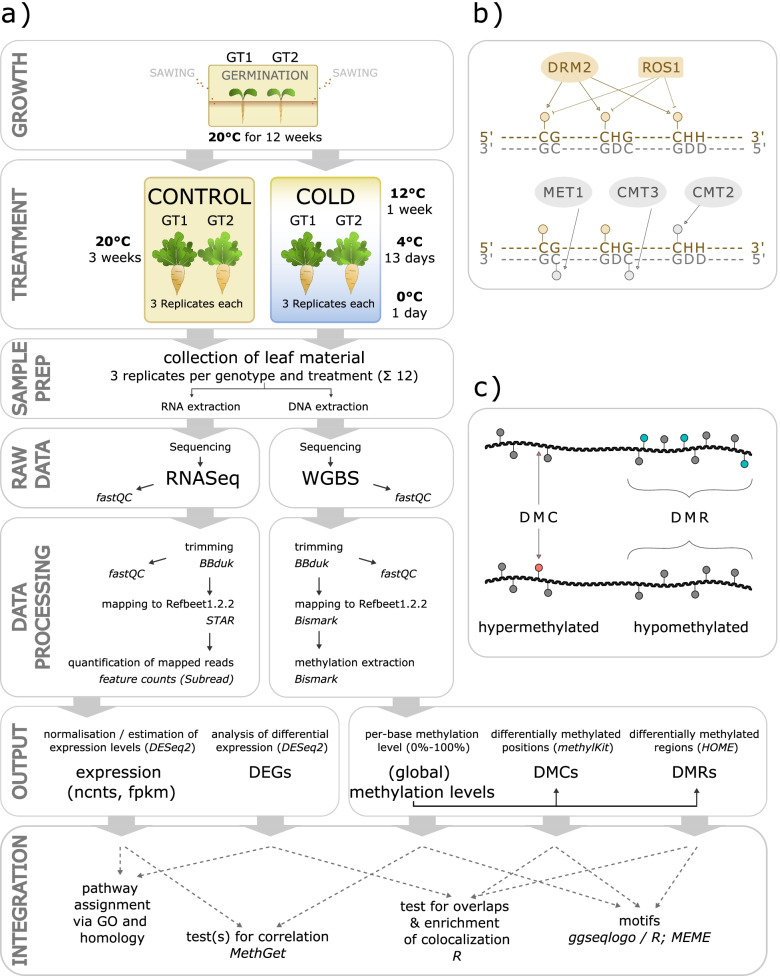
The workflow and terminology used to analyze and describe cold-dependent differential methylation in sugar beet. **a** Leaf material of two sugar beet accessions grown under control conditions or exposed to cold with three replicates per genotype and growth condition was collected. Sample material was split for extraction of RNA, or DNA, and subsequent RNA- (RNA-seq), or whole-genome bisulfite sequencing (WGBS), respectively. Raw reads were trimmed and the cleaned reads were mapped to the reference genome (Refbeet-1.2.2; [22]). For transcriptome analysis, mapped reads were quantified on gene level and the extracted data used to identify genes, differentially expressed (DEGs) between control and cold conditions. For methylome analysis, the number of methylated and unmethylated reads was extracted for each cytosine covered by at least 8 reads in each sample. From this data, methylation levels were calculated [methylated reads / (methylated + unmethylated reads)] and further analyzed in order to identify cold-dependent differential methylation affecting single cytosines (DMCs), or occurring along longer stretches of DNA (differentially methylated regions = DMRs) comprising at least three cytosines assigned to the same sequence context. To integrate transcriptomic and methylomic data, DEGs were functionally classified to identify cold-regulated, putative facilitators of DNA methylation, or DNA demethylation. Further, correlations between gene expression and methylation levels, or of changes in expression and changes of methylation were analyzed. **b** Schematic representation of context-specificity of DNA methyltransferases and demethylases. **c** Schematic representation of terms used to describe hypo (blue), or hyper (red) methylation, and differential methylation of individual positions (DMC) or differentially methylated regions (DMRs)

Plants, as sessile organisms, are known to use several strategies to cope with changing environmental conditions through significant alterations in gene expression, or epigenetic processes to ensure phenotypic plasticity. One epigenetic key mechanism is DNA methylation, which forms 5-methylcytosine nucleotides (5-mC) by covalently linking methyl-group(s) to specific cytosines at carbon position 5. Cytosine methylation in plants is facilitated by different enzymes acting on cytosines dependent on their 3′ nucleotide environment, with distinct methyltransferases being able to modify cytosines in CpG, CHG, or CHH context, where H represents A, T, or C (Fig. 1b). Sites in CpG and CHG context are considered “symmetric”, as the opposite strand inevitably carries an identical motif including a potential methylation target, which is represented by the guanine-pairing cytosine of the opposite strand [5]. Cytosines in CHH context, or methylation thereof, accordingly is “asymmetric” by nature. Apart from acting specifically towards different cytosine environments, the particular set of enzymes modulating DNA methylation is also dependent on whether methylation is being established de novo, or whether an existing methylation mark is being maintained [6]. De novo methylation is established through the RNA-directed DNA methylation (RdDM) pathway and affects cytosines in all contexts (CG, CHG, CHH). However, newly established modifications at symmetric sites would not be carried over to the daughter strand during DNA replication without an additional maintenance system. A set of distinct methyltransferases, counteract the passive loss of methylation in a context-specific manner, whereby METHYLTRANSFERASE 1 (MET1) maintains CG methylation [7, 8], whereas CHROMOMETHYLTRANSFERASE 3 (CMT3) or CMT2 maintain methylation of cytosines in CHG context [9–12]. CMT3 acts in presence of specific histone modifications (H3K9me2), placed by SUPPRESSOR OF VARIEGATION 3–9 HOMOLOGUE PROTEIN 4 (SUVH4), SUVH5, and SUVH6, which in turn are recruited to (CHG-) methylated DNA, thereby forming a reinforcing loop between CHG methylation and H3K9 methylation [13]. CHH methylation is mediated chromatin-specifically - either by DRM2-dependent RdDM (euchromatin), or - in H1-containing heterochromatin where RdDM is blocked - by CMT2 [11, 14, 15]. Cytosine methylation can also be actively removed via excision of the methylated nucleotide, followed by repair of the cleavage site (base excision repair pathway, BER). Excision of a methylated nucleotide can be facilitated by bifunctional 5-mC DNA glycosylases, such as REPRESSOR OF SILENCING 1 (ROS1), TRANSCRIPTIONAL ACTIVATOR DEMETER (DME), or DEMETER-LIKE PROTEINs (DML2 and DML3). Following further modification of the cleavage site, the gap is (filled and) repaired via DNA polymerase and ligase enzymes, e.g. LIG1 in Arabidopsis [16–21].

RNA sequencing has already been used in numerous studies to investigate altered gene expression under environmental stress in plants [23]. In addition, epigenetics relating to adaptive changes in plants gain more and more importance. Especially, the application of whole-genome bisulfite sequencing (WGBS) brings expansion of knowledge about the epigenetic mechanisms controlling the development and adaptation of many plants. The treatment with bisulfite converts unmethylated cytosines into uracil while methylated cytosines stay unaffected, which allows the detection of each cytosine’s methylation status. Genome-wide cytosine methylation analysis in *A. thaliana* already revealed methylation patterns in genes and repetitive areas [24, 25]. In crops, this technique has also been used to identify epigenetic mechanisms that have a significant impact on stress responses or developmental processes [26–30]. Many other publications also have demonstrated the importance of DNA methylation in different plants concerning responses to abiotic stress, including salt, drought, cold, heat, and heavy metal exposure [31–40]. Hébrard et al. [41] already reported genotype-dependent DNA methylation and their association to the expression of genes participating in bolting tolerance in sugar beet by comparing bolting-sensitive and bolting-tolerant genotypes. Further, Zakrzewski et al. [42, 43] published insights into the epigenome of sugar beet leaves and callus, focusing on methylation alterations at satellite DNAs and transposable elements. To improve our current understanding of cold-adaptation mechanisms in sugar beets, we investigated DNA methylomes of two *B. vulgaris* genotypes under normal conditions and after exposure to cold, focusing on conserved epigenetic alterations and their association to genome-wide gene expression. Our work provides insights into genome-wide, genotype-independent transcriptomic and epigenomic responses of *Beta vulgaris* to cold exposure and links the expression of putative methylation- or demethylation-related genes to alterations in DNA methylation.

## Results

### Experimental setup and workflow

As depicted in the overview of our workflow (Fig. 1), we used plants from two different sugar beet accessions that were either grown under control conditions for 15 weeks (from now on, corresponding samples are grouped under the label CONTROL), or for 12 weeks followed by a three-week incremental cold treatment ending at a minimum temperature of 0 °C (referred to as COLD). Leaf material was collected from six samples (2 genotypes × 3 biological replicates each) per condition (see Methods). From this material, DNA and RNA was extracted to subsequently analyze both the methylomic and transcriptomic cold responses (Fig. 1a).

### Cold treatment reduces DNA methylation levels

The 12 DNA samples were subjected to directional WGBS (PE150 + 150) using the Illumina NovaSeq 6000 platform (Novogene, Beijing, China). After adapter and quality trimming (Q20), an average of 90 million paired-end reads (150 + 150) (~ 27 Gb) per sample were obtained, representing 36-fold coverage of the sugar beet reference genome [22]. Mapping and deduplication revealed average proportions of uniquely mapped and multi-mapped reads of 60 and 12%, respectively (see Fig. 1a and Methods).

**Fig. 2:**
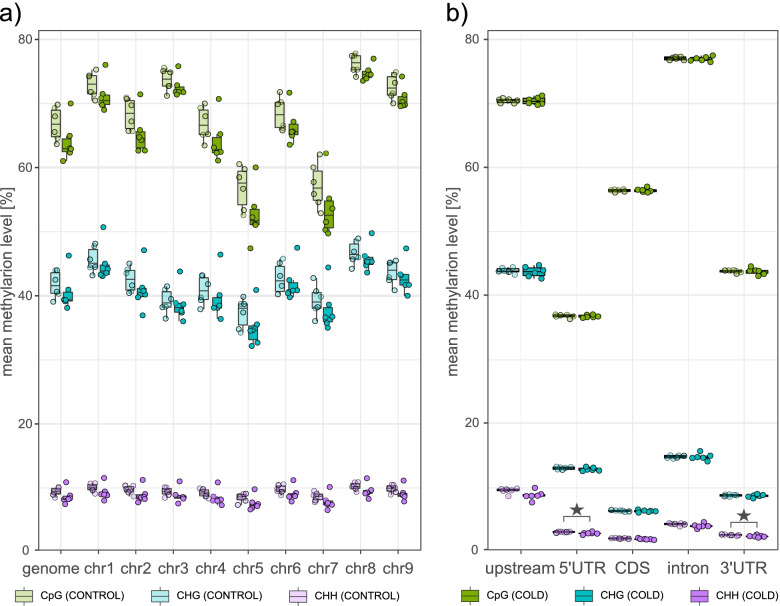
Methylation levels in the genome and in gene components under control conditions or after exposure to cold. Global methylation levels of individual chromosomes, the entire genome (panels on the left), or within gene components (panels on the right). Different colors indicate methylation levels of cytosines in different sequence contexts (CpG, CHG, CHH), with lighter shades representing methylation levels of CONTROL and saturated shades representing those of COLD, respectively. Asterisks indicate significant differences (*p*-value ≤ .05; two-tailed Welch’s test) between methylation levels of CONTROL and COLD. (See also: Additional File 1, Supplementary Fig. S1 for feature methylation levels per chromosome, and Additional File 1, Supplementary Fig. S2 for methylation levels of transposable elements)

After methylation extraction, the proportions of methylated cytosines in each context (CpG, CHG, and CHH) were analyzed per chromosome and in the genome as a whole (Fig. 2a). For beets grown under control conditions, the highest methylation levels were detected for cytosines in CpG context (mCpG: 66.8%), followed by mCHG (41.7%) and mCHH (9.4%). Cold treatment slightly decreased global methylation levels of cytosines in all sequence contexts, with methylation levels reduced by 2.8% (mCpG: 64%), 1.2% (mCHG: 40.5%), or 1.3% (mCHH: 8.7%) in the cold treated group, although differences were not statistically significant in any case (two-tailed Welch’s test; Fig. 2a). A trend towards cold-dependent demethylation was also detected on gene-feature-level. To describe these methylation patterns in the different parts of a gene, we averaged the methylation proportions for each possible context (among all 6 samples in the corresponding group) in the following regions of all annotated genes of the reference genome [22, 44]: an upstream region spanning 2.5 kb in 5′ direction of the transcription start site (TSS); 5′-untranslated region (UTR); coding sequence (CDS), intron and 3’UTR. As shown in the right panel of Fig. 2, methylation levels generally reached maxima upstream of genes and within introns. In detail, mCHG and mCHH levels peaked extragenically, i.e. in upstream regions, while maximum mCpG was detected along introns. Overall, CDSs and 5’UTRs showed comparatively low methylation levels in all contexts, with mCHG and mCHH reaching a minimum in CDSs, and lowest mCpG detected for 5’UTRs. Although COLD showed slightly lower average methylation than CONTROL in almost all gene-related features (i.e. in all categories including 5′ flanks) and for all contexts analyzed, these differences were mostly rather subtle, while statistically significant differences were detected only between 5′- or 3′- UTR mCHH levels of COLD vs CONTROL (two-tailed Welch’s test, *p* < .05).

### Exposure to cold alters methylation of individual cytosines in all sequence contexts

**Fig. 3:**
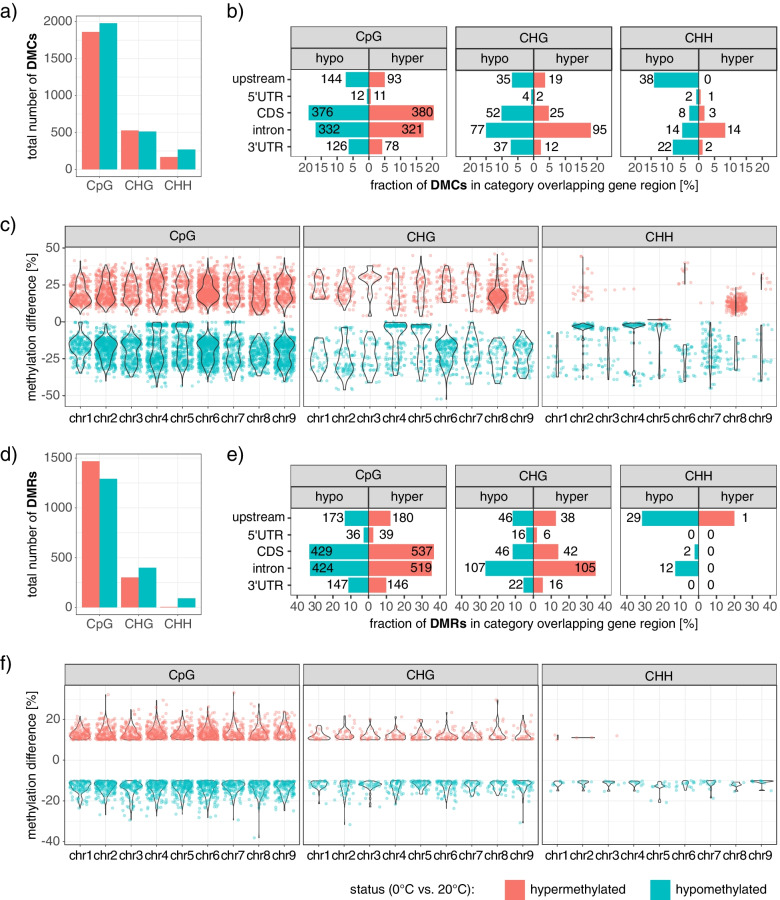
Distribution and characteristics of differentially methylated cytosines (DMCs), or differentially methylated regions (DMRs). **a** & **d** Total numbers of all hyper- (red) or hypomethylated (cyan) DMCs (q-value ≤ .05; panel **a**) or DMRs (with a minimum absolute methylation difference of 10% between CONTROL and COLD; panel **d**). **b** & **e** Fraction of hypermethylated (red) or hypomethylated (cyan) DMCs (**b**), or DMRs (**e**) in CpG, CHG or CHH context overlapping an upstream region (2.5 kb upstream of TSS), 5’UTR, CDS, intron or 3’UTR. Percentages (x- axis) refer to the fraction of associated DMCs (or DMRs) compared to all DMCs (or DMRs) of the same context and the same direction of change in methylation, respectively. Numbers indicate the count of differentially methylated positions or regions constituting the corresponding bar. **c** & **f** Distribution of differential methylation at single positions (**c**) or extending over multiple cytosines within the same sequence context in larger DNA stretches (**f**) over the chromosomes of *Beta vulgaris*. Position along the y-axis indicates the difference in methylation between CONTROL and COLD

To explore significant alterations of DNA methylation in response to cold treatment at individual positions, we analyzed methylation for each cytosine (depth 8 in each replicate) of all chromosome-assigned scaffolds constituting the *B. vulgaris* genome using methylKit [45]. A cytosine site with a q-value (SLIM adjusted *p-*value) of < .05 was defined as differentially methylated (DMC = differentially methylated cytosine), with positions being labeled “hypermethylated” (e.g. dmCpG_hyper_) when methylation rates were significantly higher, or “hypomethylated” (e.g. dmCpG_hypo_) when methylation rates were significantly lower in cold-treated samples compared to the controls (Fig. 1c). We identified 5319 DMCs distributed over the nine chromosomes (Fig. 3a), with the highest DMC-density on chromosome 6 (19.7 DMCs per Mbp), and the lowest on chromosome 1 (7.5 DMCs per Mbp; Fig. 3c). 3840 (72.2%), 1041 (19.6%) and 438 (8.2%) DMCs could be assigned to CpG, CHG, and CHH, respectively, with slightly higher numbers of dmCpG_hypo_ and dmCHH_hypo_ compared to corresponding hypermethylated Cs (Fig. 3a). Of all detected DMCs, 2873 (54.0%) were either located directly within, or occurred within 2.5 kb 5′ of TSSs, with dmCpG accounting for more than 80% of all DMCs overlapping a gene or its upstream flank, approximately reflecting the proportion of DMCs in CpG context to non-CpG-DMCs.

Overall, most DMCs overlapped introns, CDSs, or occurred within 2.5 kb upstream of TSSs - together accounting for more than 90% of all gene-associated or 5′-proximal DMCs (Fig. 3b), except for dmCHH_hypo_, which showed a preference for upstream regions and 3’UTRs. Additionally, dmCHG_hyper_ and dmCHH_hyper_ showed particularly strong associations with introns. Note, that while this was beyond the scope of our study, we additional provide corresponding data summarizing overlaps of DMCs (and DMRs, see next chapter) with transposable elements (TE) in Additional File 1, Supplementary Fig. S2 and S3. In plants, methyltransferases and demethylases favor specific sequences for local (de)methylation [46]. To evaluate possible sequence preferences, we scanned the sequence composition within 25 bp-flanking-regions (25 bp up- and downstream of each DMC, i.e. 51 bp in total) of all cold-dependent DMCs, separately for hyper- and hypomethylation in CpG, CHG, or CHH context, respectively (see Additional file 1, Supplementary Fig. S4). In flanking regions of dmCHG and dmCpG, we observed mainly A/T rich sequences, with a slightly higher frequency of Gs 5′ proximal to dmCHG (position − 4 relative to DMC position). In contrast to dmCpG and dmCHG, Gs and Cs occurred more frequently in the neighborhood of dmCHH. Within trinucleotide-environments of hypermethylated DMCs, H positions directly next to differentially methylated cytosines were almost exclusively occupied by T or A. Some of the cytosines which had instead lost methylation in response to cold treatment preceded another (not necessarily differentially methylated) cytosine. These results are comparable to identified sequence preferences for methylation in *A. thaliana* [24, 25].

### Differentially methylated regions in CHH context preferentially become hypomethylated in response to cold

Differentially methylated regions (DMRs) are stretches of DNA including multiple cytosines with collectively altered methylation levels between samples. We used HOME (Histogram of methylation), a machine learning-based tool, to identify DMRs while taking into account the sequencing depth and spatial correlation of cytosines [47]. We decided to filter all detected differentially methylated regions for a minimum methylation difference of 10% (spanning the whole DMR) between CONTROL and COLD. This left a total of 3557 DMRs (Fig. 3d), with 2760 DMRs (77.6%) in CpG, 700 (19.7%) in CHG, and 97 (2.7%) in CHH context. 699 DMRs (19.7%) - all of which were assigned to either CpG or CHG - contained at least one DMC (in corresponding C context). We investigated the presence of DMRs within gene features, and – again similar to DMCs - the number of DMRs in CpG context was highest in CDSs and introns and lowest in 5’UTRs (Fig. 3e). DMRs in CHG context were similarly distributed over gene features, but peaked within introns, both contrasting CHH DMRs, which predominantly overlapped upstream regions but – apart from some introns - were basically absent from other gene features. Comparable to DMCs, most of the DMRs were located on chromosome 6, while chromosome 1 showed the fewest DMRs (Fig. 3f).

### CHG and CHH methylation is associated with low gene expression

For RNA-seq, RNA extracted from all six CONTROL and all six COLD samples was used to construct poly-A selected libraries and sequenced using the Illumina Inc. HiSeq 2000 system (GATC GmbH, Konstanz, Germany). After adapter and quality trimming (Q20), an average of 88 million paired-end (151 + 151) reads (~ 13 Gb) per sample were obtained. The average proportions of uniquely mapped and multi-mapped reads were 91.06 and 5.81%, respectively (see Fig. 1a and Methods).

**Fig. 4:**
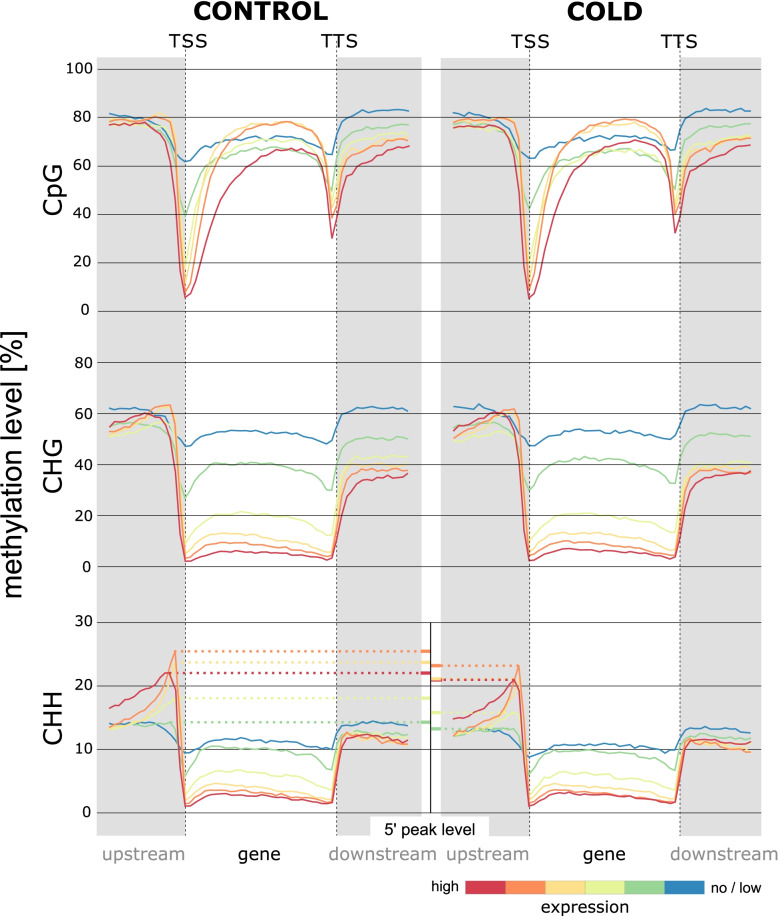
Methylation profiles along lowly or highly expressed genes. Profile plots depict data corresponding to methylation and expression in one (representative) sample of CONTROL (profiles on the left), or of COLD (profiles on the right). Genes were ranked and assigned to one of six categories (color-coded) based on their expression level (FPKM; i.e. after normalizing for gene lengths) in a given sample. All genes were split into 30 windows (TSS to TTS, including introns) and window-averages of all genes within the same category (i.e. with similar expression levels) are plotted as an individual line profile, overall depicting the distribution of methylation for groups of lowly, intermediate (orange to green) or highly expressed genes. Gene flanks extend another 15 windows (half the gene length) 5′ of the TSS (upstream) or 3′ of the TTS (downstream), respectively. The inset in the lower panel (between CHH profiles of CONTROL and COLD) compares peak levels of mCHH (5′ region of genes), to illustrate decreased mCHH levels of the COLD sample throughout different expression categories

Integration of gene expression and DNA methylation additionally requires consideration of sequence contexts of cytosines while discriminating between different types of genomic features affected by methylation. We used MethGet to assess associations between expression and methylation for each replicate, individually [48]. Gene expression data was normalized for transcript length to allow for the comparison of gene expression within samples. Each gene was assigned to one of six groups, based on its relative expression level. Subsequently, the methylation level along all genes within the same group, as well as of their flanking regions (spanning about half the length of a given gene 5′ of the TSS, and half the gene-length 3′ of the TTS [transcription termination site]) was evaluated. As shown in Fig. 4 (also see Additional File 1, Supplementary Fig. S5), the highest methylation levels were reached in flanking regions, particularly in those upstream the TSS, and this tendency was independent of the extent of expression, sequence context, or treatment. Generally, methylation of cytosines in CpG context showed a marked drop in a narrow region around the TSS, as well the TTS with methylation levels within genes reaching those observed further upstream of the gene. In CHG and CHH context, the methylation level also decreased when approaching TSS or TTS, but remained comparably low within genes. Interestingly, expression levels were not necessarily associated with the total gene methylation level of cytosines in CpG, but rather with the amplitude of the drop in methylation near the TSS, with highly expressed genes showing the steepest decline, i.e. the lowest methylation levels around the TSS and lowly expressed genes showing the lowest deviation from upstream- and within-gene regions and thus the highest methylation level in proximity to their TSS. In addition, expression of the corresponding genes seemed to be negatively correlated with the methylation levels of CHG and CHH along the entire length of the gene. There, high expression was accompanied by low methylation levels throughout, whereas methylation of lowly expressed genes was higher, approximating upstream and downstream methylation levels. In general, methylation profiles were almost identical between CONTROL and COLD. However, closer inspection revealed small but noticeable differences between 5′ mCHH peak levels of CONTROL and COLD samples (indicated in Fig. 4, CHH panel), with gene groups of COLD samples reaching slightly lower maximum methylation levels than gene groups with comparable expression levels of CONTROL samples. But because gene groups are reconstituted for each sample individually and thus their composition partially differs between samples, an effect of 5′ mCHH levels on gene expression cannot be inferred from this observation.

### Cold triggers genotype-independent changes in gene expression

**Fig. 5:**
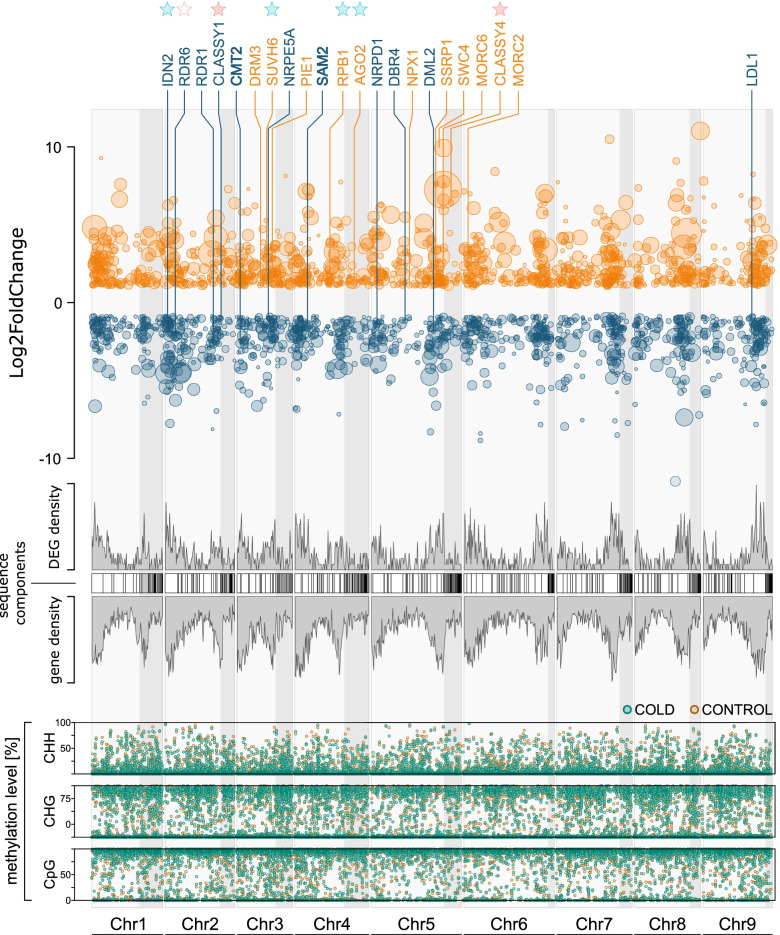
Differential gene expression in *Beta vulgaris* in response to cold exposure. Scatterplot depicting changes in expression (Log2FoldChange) and chromosomal positions for all differentially expressed genes (DEG; padj ≤ .05, Log2FoldChange ≥1 or ≤ − 1) between CONTROL and COLD. Genes, whose expression was significantly upregulated in cold-treated sugar beets compared to the control, are depicted in orange. Those whose expression was significantly downregulated, are depicted in blue. Dot size indicates significance (padj), with larger dots representing higher significance (i.e. lower padj). Labels indicate DEGs related to DNA methylation or demethylation. Densities (grey) correspond to those of all DEGs, or of all predicted genes [44, 50] in 0.5 Mbp windows. Sequence components constituting (pseudo-) chromosomes of the reference sequence (RefBeet-1.2.fna.gz, [22] downloaded from ‘The *Beta vulgaris* Resource’, [50]) are shown as blocks, with white blocks representing localized scaffolds (known position and orientation within chromosome) and grey boxes representing unlocalized scaffolds (assigned to a chromosome but precise position and/or orientation unknown), the latter being shaded in light grey over the whole plotting area. Asterisks highlight (de)methylation related DEGs overlapping with hyper- (red) or hypomethylated (cyan) DMRs (filled) or DMCs (hollow) – all overlapping DMRs or DMCs could be assigned to cytosines in CpG context. Lower panels show average methylation levels of CONTROL or COLD at 1000 randomly selected cytosines per chromosome and context

Analysis of differential expression [49] contrasting CONTROL vs. COLD yielded in total 2549 DEGs (Additional File 2), most of them localized on chromosome 6, fewest on chromosome 8 (Fig. 5). Of them, 1244 were up-regulated, while 1305 were down-regulated in COLD (Wald-test; padj ≤.05; |log_2_FC| ≥ 1).

### Differential methylation does not globally correlate with gene expression

**Fig. 6:**
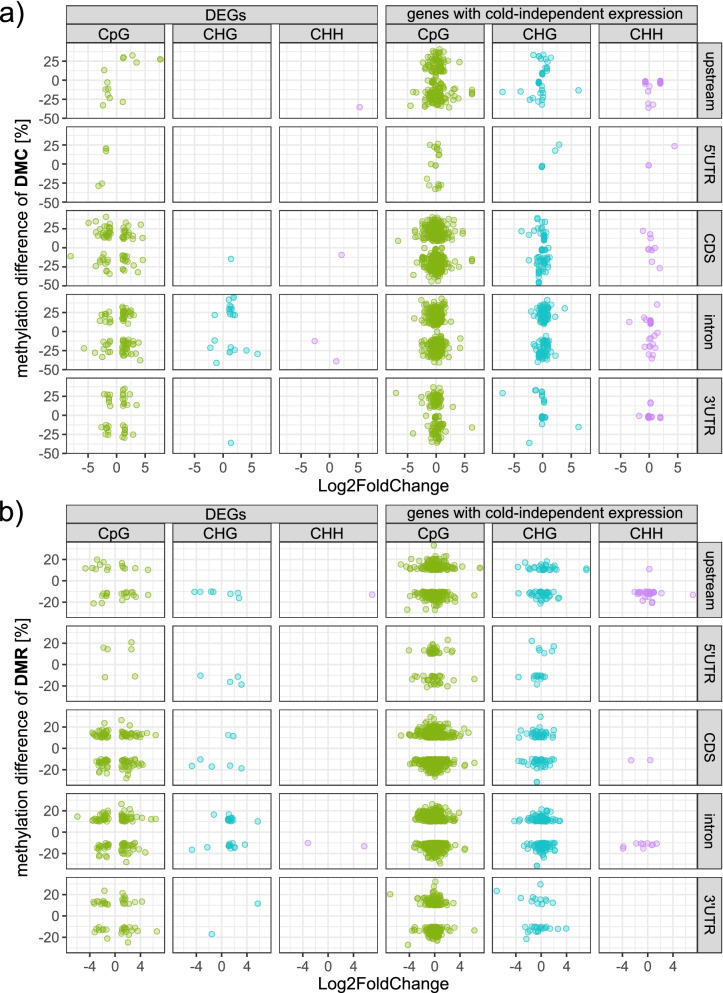
Association between changes in expression and changes in methylation. **a** & **b** Plots depicting correlations between changes in methylation (methylation difference) and changes in expression (Log2FoldChange), for (**a**) DMCs or (**b**) DMRs overlapping an annotated gene. Panels on the left show association of DMCs/DMRs with DEGs, panels on the right correspond to DMC−/DMR-overlaps with genes, whose expression was not significantly altered by cold. Different colors indicate differential methylation in the three sequence contexts, with CpG in green, CHG in turquoise and CHH in purple

We identified 209 differentially expressed genes (from a total of 2549 DEGs; ≙ 8.2%) that were either directly overlapped by one or more DMCs, or had at least one DMC co-localizing with their 2.5 kb upstream regions. Comparing the proportion of DMC-associated genes among the DEGs (8.2%) with the fraction of DMC-associated genes (again with gene-ranges extended to comprise an additional 2.5 kb 5′ of TSSs) among all annotated genes (6.6%, corresponding to a total of 1712 DMC-associated genes), association with differential methylation was significantly enriched among our set of DEGs [χ^2^(1, *n* = 26,004) = 4.9091, *p* < .05]. However, combinations of hyper- or hypomethylation and up- or downregulation of expression did not reflect an apparent tendency (Fig. 6a): from the total of 1244 transcriptionally upregulated genes, 66 were overlapped by at least one hypermethylated DMC, while similarly, 62 were overlapped by at least one hypomethylated DMC. Analogously, of the total of 1305 genes that were significantly downregulated by cold, 46 DEGs colocalized with hyper-, and 43 DEGs colocalized with hypomethylated DMCs, respectively. DMCs preferentially occupied CDSs, introns, and 3’UTRs of DEGs, independent of the sequence context of the DMC. For genes showing unaltered expression following cold exposure, this pattern shifted from 3’UTRs towards upstream regions (Fig. 6a).

Similarly, 260 DEGs coincided with at least one of the total of 3557 regions (DMRs) where methylation levels differed by at least 10% between CONTROL and COLD. The relative distribution of these DMRs within the affected DEGs was similar to those observed for DMCs, i.e. DMRs preferentially occurred within CDSs, introns, and 3’UTRs of DEGs (Fig. 6b). Of all genes, 2287 (≙ 8.8%) showed association with a DMR, and comparing the absolute numbers, DEGs were again enriched with DMRs [χ^2^(1, *n* = 26,004) = 28.9613, *p* < .01]. This could lead to the conclusion that the methylation status of our set of DEGs dynamically changed not only at single positions (DMCs) but also in extended areas of methylation alterations over multiple cytosines (DMRs) as a response to cold treatment.

### Altered expression of genes involved in DNA (de)methylation correlates with observed methylation changes

**Fig. 7:**
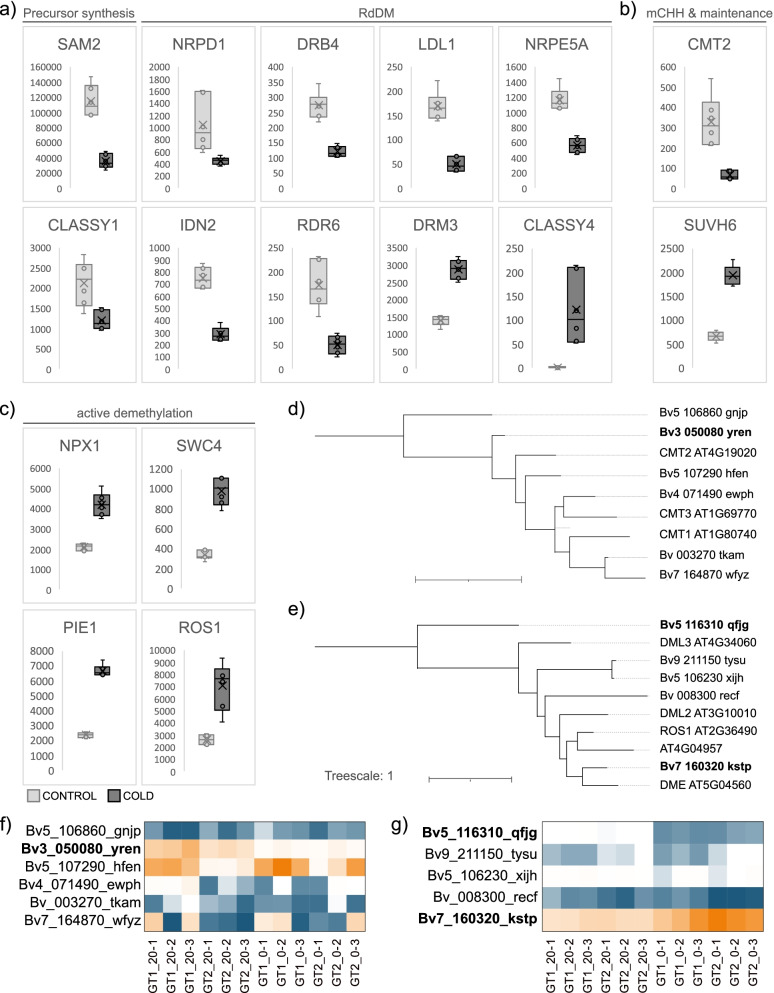
Expression of DNA methylation or demethylation-related DEGs between CONTROL and COLD. **a-c** Normalized counts of differentially expressed genes putatively involved in precursor synthesis (SAM2) or RdDM (**a**), maintenance or de novo CHH methylation (**b**), or active demethylation (**c**). Boxes in light grey represent values for all samples in CONTROL, dark grey represents values for all samples in the COLD-treated group. **d-e** Phylogenetic analysis of chromomethyltransferases (**d**) or bifunctional nucleases (**e**) of *Beta vulgaris* and *Arabidopsis thaliana*. **f-g** Heatmaps depicting transcript levels (DESeq2 normalized counts) of CMT-related (**f**) or DME/ROS-related (**g**) genes in all samples of CONTROL and COLD with high values in orange and low values in blue (white represents the median of normalized counts for genes depicted in the same heatmap). Bold geneIDs indicate differential expression between CONTROL and COLD (adjusted *p*-value ≤ .05, |Log2FoldChange| ≥ 1])

**Fig. 8:**
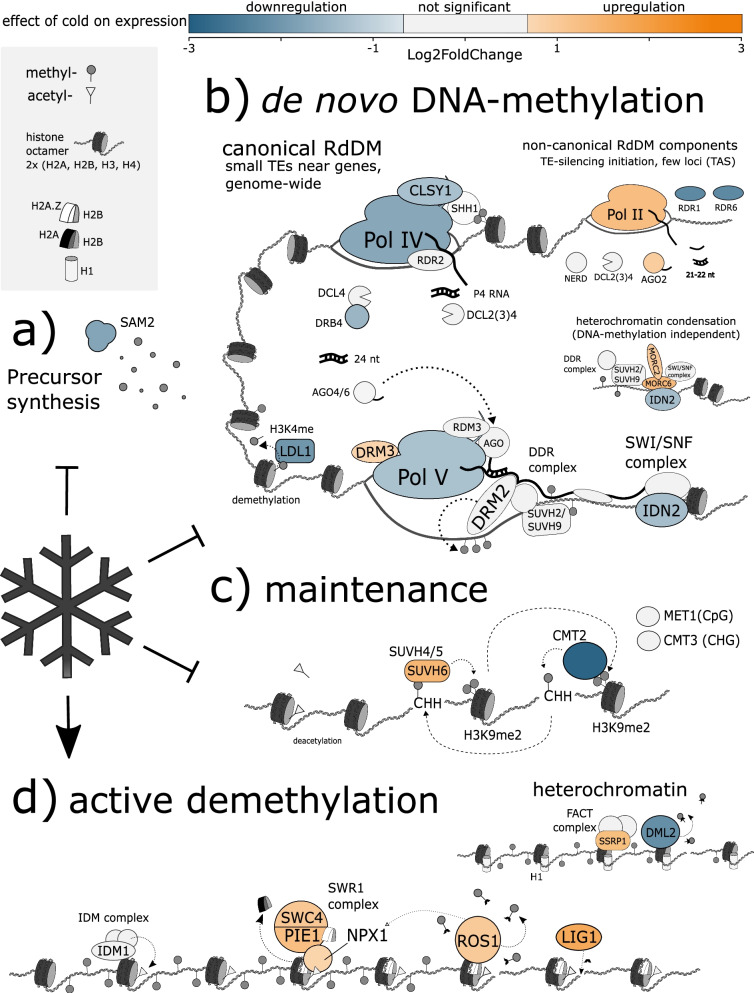
Cold reduces DNA methylation via upregulation of genes within the ROS1-pathway. Model depicting the observed transcriptional effect of cold treatment on expression of sugar beet homologs of DNA methylation- or demethylation-related genes **a**) mediating synthesis of s-adenosylmethionine or acting within **b**) the RdDM, **c**) methylation maintenance, or **d**) DNA demethylation pathways. The schematic illustration depicts differentially expressed sugar beet homologs of Arabidopsis genes, known to participate in the corresponding pathways as colored objects (blue to orange). (Homologs of) Additional genes contributing to each pathway, which were not differentially expressed in sugar beets in response to cold or for which no sugar beet homolog was identified, are shown as black-and-white structures to provide an overall representation of the entity of known players mediating the depicted mechanisms. Labels (corresponding to the gene name of each Arabidopsis homolog) and corresponding sugar beet accessions are provided for all DEGs in Additional File 2. For detailed functional description of the depicted genes and pathways, see main text

Vice versa, we systematically screened our set of DEGs for known players involved in DNA (de)methylation and mapped the matching DEGs to general pathways known to affect DNA methylation. We found numerous genes whose homologs have been described to contribute to either a) de novo methylation via RdDM, b) chromomethyltransferase-mediated maintenance (or - for CHH - de novo establishment) of DNA methylation or c) active DNA demethylation (see Additional File 2 and section Functional classification and phylogenetic analysis in Methods for details on the classification, annotation and pathway assignments of DEGs) and whose expression was significantly altered in response to cold (see Fig. 7 a-c for expression levels of these genes in CONTROL or COLD, respectively; and see Fig. 8 for a schematic model depicting pathways, in which the highlighted DEGs are predicted to interact with additional [non-deregulated] genes to eventually drive alterations of DNA methylation in response to cold). Cold treatment significantly reduced expression levels of subunits of DNA-directed RNA polymerase IV (*NRPD1*; *Bv5_101150_qmjs*) and DNA-directed RNA polymerase V (*NRPE5A*; *Bv3_062710_wdck*), possibly resulting in reduced production of siRNAs and scaffold RNAs for RdDM. In line with this, chromatin remodeler SNF2 DOMAIN-CONTAINING PROTEIN CLASSY 1 (*CLSY1*; *Bv2_044610_uypz*), an interactor of Pol IV, as well as several RNA-dependent RNA polymerases (*RDR1*, *RDR3*, *RDR6*, corresponding to *Bv2_040860_udas*, *Bv8_197280_tuhe*, and *Bv2_030230_aisi*, respectively), which are thought to produce dsRNA from mainly Pol IV-derived templates, were significantly downregulated, as was the expression of *DRB4* (*Bv5_109930_mrpd*), which is thought to assist the processing of dsRNA fragments to siRNA via DCL4 [51, 52]. Furthermore, cold significantly decreased the expression of *IDN2* (*INVOLVED IN* DE NOVO *2*; *Bv2_024880_hakm*) - an interactor of DRM2 required for RdDM - as well as of a homolog of DNA (cytosine-5)-methyltransferase *CMT2* (*Bv3_050080_yren*, also see Fig. 7d and f), which mediates de novo methylation preferentially at cytosines in CHH context [15, 53]. However, phylogenetic analysis suggests that another gene, whose expression was not cold-dependent nor consistent between genotypes (Fig. 7d, f), but which showed partially high expression (comparable to *Bv3_050080_yren*), might act as the actual functional homolog of CMT2 at least in one of the genotypes, or act redundantly to the DEG candidate, *Bv3_050080_yren*. Finally, cold-dependent transcriptional downregulation was also observed for an *S-adenosylmethionine synthase* (*SAM2*; *Bv4_079640_yozf*), whose product acts as a major methyl-donor in DNA methylation. Together, these transcriptional changes overall indicate a reduction in RdDM derived de novo methylation and decreased CMT2-based CHH methylation maintenance. In addition, we detected several transcriptional changes that might indicate enhancement of active DNA demethylation upon cold treatment: the cold-triggered increase of *NPX1* (*Bv5_110670_yfki*) expression, together with enhanced expression of the SWR1-components *PIE1* (*Bv3_065010_faku*) and *SWC4* (*Bv5_122550_anjp*) could promote the recruitment of ROS1 via deposition of H2A.Z. Furthermore, the expression of *ROS1* (*Bv7_160320_kstp*) itself was upregulated upon cold treatment. Phylogenetic analyses and the almost complete lack of expression of other sugar beet homologs from the same gene family (Fig. 7e and g) contributed to the functional classification of this gene as the main ROS1-homolog. (Note, that a *DNA LIGASE 1-LIKE* homolog, *Bv_001710_ogue*, was additionally upregulated more than four-fold. However, for conciseness, we excluded this gene from global analyses due to its location on a sequence component not assigned to any chromosome.) Expression of a homolog of the putative DNA glycosylase At3g47830 | DEMETER-like protein 2 (*DML2*; *Bv5_116310_qfjg*) was significantly lowered by cold (Fig. 7).

## Discussion

### Limitations

The empirical results reported here should be considered in the light of some limitations. A large proportion of the genome of *Beta vulgaris* (42.3% of the Refbeet assembly, [22]) consists of repetitive sequences, rendering it a challenging target particularly during read mapping. Furthermore, the same reference was used to map reads to - irrespective of the treatment. This means that potential genomic rearrangements (e.g. transpositions) that had occurred as a response to the applied stress, are inevitably missed during the analysis. This is mainly relevant for analyses of WGBS data, which (as opposed to RNA-seq) theoretically covers the entire genome. Retrotransposons are the most abundant type of repetitive elements identified in Refbeet1.2.2 [22], and activation as well as transposition, in fact, has been reported to be triggered by abiotic stresses, including cold [54, 55]. Although this was beyond the scope of our study, and has been examined in detail in earlier studies [42, 43], we detected significant decrease of mCHH along several retrotransposon subtypes (mainly DNA and LTR, Additional File 1 Supplementary Fig. S2 and S3), which could potentially be linked to transcriptional activation of transposons or nearby genes. The fact that we used Refbeet1.2.2 as reference, which is based on an independent sugar beet accession (KWS2320, [22]), further complicates proper alignment of reads, as well as interpretation of WGBS data. Galewski and McGrath [56] recently examined lineage-specific variation (LSV), comparing closely related *Beta vulgaris* crop-types and different *Beta vulgaris* accessions of the same subspecies between or within groups. With about 0.23%, the described LSV between accessions of the same subspecies (sugar beet) was low compared to the high variation between crop types (99.37%) - as was the variation observed within other crop type lineages (approximately 0.40%). A comparatively large fraction of variation distinguishing different sugar beet accessions, however, was concentrated on chromosome 6, which could be due to drift or divergent selection on this chromosome. It is conceivable that the ability to change methylation status at this chromosome in response to the environment could contribute to a fine-tuned regulation of breeding-relevant genes. And this would be consistent with the observed high DMC density on chromosome 6 (Fig. 3c) following cold exposure. However, genetic variation (distinguishing the accessions used in this study from the reference) can affect the outcome of WGBS analysis, for one, because it negatively impacts mapping efficiency, and secondly, because methylation is eventually determined based on comparison of the read to the reference. This means that a C (reference genome) to T mutation present in both accessions, is eventually scored as a non-methylated cytosine (which are converted to T during bisulfite treatment). In turn, for positions representing a T to C mutation, where the C could be methylated or non-methylated in the analyzed accessions, no methylation information will be extracted, resulting in loss of information for such positions. In the present study we mainly focused on the investigation of genotype-independent adaptation of *Beta vulgaris subsp. vulgaris* to cold exposure. By eventually comparing control grown plants with cold treated plants of the same accession(s), genotype-dependent responses and variation should be cancelled out, at least partially. While this comes with a price, as the combination of two accessions eventually dilutes effects that are pronounced only in one of the genotypes, it should also decrease artifacts arising from variation between our accessions and the reference, for example during the examination of differential methylation between COLD and CONTROL. Yet, we did detect several low-difference DMCs (Fig. 3c) and found that the majority of them occurred highly concentrated within rather narrow sequence segments. Upon further inspection, we could assign most of these regions to areas, where coverage was highly above average. The seemingly high coverage, in turn, could be attributed to comparatively large numbers of falsely mapped reads in those regions, which represented highly repetitive sequences. More precisely, low-difference-high-coverage “DMC” clusters occurred almost exclusively within sequences annotated as or predicted to code for rRNAs (see also Additional File 1, Supplementary Fig. S2 and S3).

### Characteristics of cytosine-methylation in sugar beet

In this work, we have analyzed and compared the methylome (WGBS) and transcriptome (RNA-seq) of sugar beet under control conditions or after exposure to cold. Under control conditions, cytosine methylation proportions were 66.8% (mCpG), 41.7% (mCHG), and 9.4% (mCHH) for total sequenced CpG, CHG, and CHH, respectively (Fig. 2). This deviates from the results obtained by Niederhuth et al. [57], who reported methylation rates for *B. vulgaris* of as high as 92.5% (mCpG), 81.2% (mCHG), and 18.7% (mCHH). It might be noteworthy that the authors highlighted that, particularly the percentage of mCHH in their study, was “unusually high” and define *B. vulgaris* as a “notable outlier”. The lower methylation rates for the three analyzed contexts (mCpG, mCHH and mCHG) in our analysis, in contrast, are in more concordance with the values of other angiosperm species included in Niederhuth et al. [57] and with the methylation proportion for *B. vulgaris* detected by Zakrzewski et al. [43]. Although data was obtained from leaf material in both studies, factors such as plant age or growth conditions (neither are documented for the comparative analysis of Niederhuth et al. [57]), the use of different accessions or the fact that sequencing data was generated and processed using different methods and computational tools, might to some extent account for the differences in observations of methylation levels.

### *Beta vulgaris* fine-tunes global methylation levels in response to cold

After cold treatment, methylation proportions dropped to 64.0% (mCpG), 40.5% (mCHG) and 8.7% (mCHH) which, in all cases, represented a decrease in total methylation, although none of them was statistically significant (two-tailed Welch’s test). Previous studies have also detected low to medium but consistent variations in methylation proportions due to abiotic stresses. However, the intensity and direction of the change of hypo- or hypermethylation depends on the species, type of treatment and genotype pre-adaptation [58]. In upland cotton *(Gossypium hirsutum)*, for example, Lu et al. [37] found a decrease of 2% in mCpG and mCHG, and an increase of 4% in mCHH, after drought stress. Gayacharan and Joel [59] observed that, after drought treatment, drought sensitive rice genotypes were generally hypermethylated, whereas the drought tolerant ones were hypomethylated. Salinity stress caused DNA demethylation in *Setaria italica* [28], but increased methylation in *Medicago truncatula* [60]. Cold stress was found to trigger DNA demethylation in roots of maize seedlings [61]. Also tartary buckwheat (*Fagopyrum tataricum*) tended to decrease DNA methylation following cold shock (at 0 °C for several hours) or repeated short term (< 1 day) exposure to cold. However, mCHH was actually slightly denser in repeatedly cold-exposed plants compared to seedlings cultivated under control conditions [62]. In contrast, cucumber (*Cucumis sativus*) radicles increased DNA methylation in response to cold treatment, which was paralleled by reduced growth rates [63]. In rubber trees (*Hevea brasiliensis*), and fruits of sweet oranges (*Citrus sinensis*), or of tomato (*Solanum lycopersicum*), cold was shown to alter expression of specific genes involved in cold-response, volatile-, or pigment-synthesis through altered DNA methylation of corresponding promoters [40, 64, 65]. Finally, in some woody perennials like poplar, cold triggers DNA demethylation prior to bud break [66].

### Large amplitudes in methylation profiles distinguish highly from lowly expressed genes

mCG, mCHG and mCHH patterning has been linked to gene expression, but methylation levels can widely vary between species. In rice, mCpG is lowest at the flanks of the TSS (approximately 100 bp upstream and 500 bp downstream of TSS) and the TTS (approximately 500 bp upstream and 100 bp downstream of TTS), mCHG and mCHH are basically absent from genes [67]. Cassava, soybean and *A. thaliana* have similar methylation patterns, with almost exclusive methylation of cytosines in CpG context, which become very low around the TSSs and TTSs [24, 38, 68]. Further, mCpG at the TSS and TTS tends to inversely correlate with expression levels of the corresponding gene [67, 69]. Independent of the cytosine context, we observed the lowest methylation levels in close proximity to the TSS and TTS regions of genes and much higher levels of methylation in the 2 kb up- and downstream region of TSS and TTS, and - at least for mCpG - an intragenic increase in methylation. These profiles match those detected by Zakrzewski et al. [43], who reported very similar methylation patterns based on WGBS data from sugar beet leaf material.

We detected a genome-wide hypomethylation in response to cold treatment. This trend was quite evenly distributed over the whole genome of *B. vulgaris* with significant alterations especially in CHH context (and to some extent CHG) after cold treatment in almost all gene regions analyzed (Fig. 2; Additional File 1, Supplementary Fig. S1). Although we observed methylation in CHG and CHH context in all genes to be associated with a lower level of expression, our set of differentially expressed genes showed no obvious pattern of up- or downregulated expression and in- or decreased methylation in any sequence contexts (Fig. 4 and Fig. 6). This means that while absolute methylation levels in CHG and CHH context do, in fact, inversely correlate with gene expression (Fig. 4), changes in methylation (i.e., DMCs or DMRs) are not necessarily associated with a significant change in expression (Fig. 6).

On the other hand, we identified many homologs of known players in our set of DEGs, whose functions were already attributed to de novo- or maintenance of DNA methylation or active DNA demethylation in *A. thaliana* (also see “Functional classification and phylogenetic analysis” in “Methods” and Additional File 2) and which might explain the observed changes in methylation on a transcriptional basis.

### Differential expression of chromatin modifiers and DNA (de)methylaters correlates with changes in global methylation

#### Cold limits precursor synthesis for DNA methylation

S-adenosylmethionine acts as co-substrate and methyl-donor for several biochemical reactions, including histone- and DNA methylation (Fig. 8a). SAM (S-adenosylmethionine synthetase) catalyzes the final step in the synthesis of the molecule. Altered expression of SAMs not only affects expression of genes related to abiotic stress [70–73], but also modifies the methylation status of DNA. In detail, missense mutation of *SAM3/MAT4* in Arabidopsis was shown to result in decreased CHG and CHH DNA methylation [74]. While cold decreased transcription of a *SAM* homolog in sugar beet leaves, its product is involved in such a multitude of processes, that it is difficult to predict hypomethylation based on decreased expression of *SAM2* alone.

#### Cold alters RNA-directed DNA methylation

However, apart from precursor synthesis, cold treatment had significant impact on expression of some enzymes considered as core components of DNA methylation-, as well as demethylation-pathways.

In plants, de novo DNA methylation of cytosine residues in all contexts is established through RdDM (Fig. 8b). Canonical RdDM is initiated through the activity of DNA-directed RNA-polymerase IV [75–78], which generates short, non-coding, single-stranded transcripts at sites enriched for particular histone modifications (H3K9me2). The histone marks are recognized by SHH1, which - in the presence of CLSY1 [79, 80] - triggers binding of Pol IV [81–83]. Prior to acting as actual guide for DNA methylation, Pol IV transcripts are processed by a series of further enzymes: RNA-DEPENDENT RNA POLYMERASE 2 (RDR2) complements Pol IV transcripts into dsRNA [84–86], which can be cleaved into siRNAs by DICER-LIKE PROTEINs [87, 88], before being loaded to ARGONAUTEs (mainly AGO4 and 6 [89, 90]). At the target site, nascent transcripts produced by another core RdDM-polymerase, Pol V, can pair with appropriate AGO-coupled siRNAs [91]. This finally recruits DOMAINS REARRANGED METHYLASE 2 (DRM2), an interactor of AGO4, to methylate cytosines at the target site [92–94].

Although we observed significantly altered expression of core RdDM components, we do not believe that the substantial global reduction of methylation levels after cold treatment can be attributed to reduction of de novo methylation by RdDM: As shown in Figs. 7 and 8, cold significantly reduced expression levels of the largest subunit of Pol IV (*NRPD1*), as well as another polymerase-subunit specific to Pol V (*NRPE5A*), which together might decrease production of siRNAs and scaffold RNAs for RdDM [95–97]. In addition, expression of *CLSY1* was significantly decreased in cold-treated sugar beets, as was the expression of three *RDRs* (*RDR1*, *3*, *6;* Fig. 8b) of which at least two participate in non-canonical RdDM at stages of alternative siRNA production in Arabidopsis [98]. Furthermore, cold decreased expression of *DRB4*, which is thought to assist processing of dsRNA fragments to siRNA via DCL4 [51, 52], as well as of *IDN2* (*INVOLVED IN* DE NOVO *2*), which binds dsRNA, associates with DRM2 and is required for recruitment of SWI/SNF chromatin remodelling complex components to RdDM target sites [99, 100]. It is thought to normally promote DRM2-mediated DNA methylation at some target loci [101, 102].

However, expression of the *DRM2*-homolog, *DRM3*, was significantly upregulated by cold. But while DRM3 is thought to regulate DRM2-mediated DNA methylation and to be required for normal maintenance of non-CpG methylation, DRM3 itself lacks a conserved site required for methyltransferase activity and compared to *drm2* mutants, Arabidopsis plants lacking functional DRM3, only show moderate losses of DNA methylation [103–105]. Additionally, some of the most crucial (canonical) RdDM components, e.g. RDR2, DCLs, AGO4 or AGO6 and, most importantly, the major RdDM DNA methyltransferase, DRM2, remained stably transcribed in cold-treated plants (Fig. 8b). And while AGO2 and Pol II – both of which were upregulated in cold-treated samples (Fig. 8b) - can be involved in providing siRNAs that eventually guide DNA methylation via DRM2 through non-canonical, partially Pol IV-independent mechanisms of RdDM [98], the protein products of both genes mainly mediate other regulatory mechanisms not necessarily affecting DNA methylation.

Depending on the target and scaffold RNAs involved, RdDM can facilitate methylation of several consecutive cytosines and thus, presence of hypermethylated DMRs could hint towards increased RdDM at these positions. In our data, there was a higher number of individual CpG sites showing significant hypomethylation, than there were hypermethylated CpG positions (Fig. 3a). In contrast, among longer stretches of DNA that showed considerable (> 10%) cold-dependent changes in CpG methylation (i.e. DMRs in CpG context), the majority had actually gained CpG-methylation upon cold exposure (i.e. more hyper- than hypo-methylated CpG-DMRs; see Fig. 3d). DRM2 is able to methylate cytosines independent of their sequence context [92, 106]. Therefore, DMRs in a particular sequence context which have gained methylation through RdDM (i.e. through DRM2), are expected to show overlap - at least to some extent - with hypermethylated DMRs in other sequence contexts. However, although there were about 100 CpG-DMRs that coincided with CHG-DMRs and showed matching trends in their methylation change (both overlapping DMRs either gained methylation, or both exhibited loss of methylation, but not a combination of both), about half of them were hypomethylated DMRs. The remainder, i.e. overlapping hypermethylated CpG/CHG DMRs might indeed represent regions with locally enhanced RdDM activity.

Overall, while the altered expression of several RdDM- related enzymes might indicate a change in RdDM, our observations do not support the conclusion that loss of RdDM is the major determinant of the global reduction in methylation after cold treatment.

#### Downregulation of CMT2 suggests hypomethylation in CHH

Generally, when or if mCpG, mCHG and/or mCHH are established, these methylation marks must be actively maintained in order to retain correct DNA methylation patterning (Fig. 8c). Otherwise, methylation information gets lost on the daughter-strand during DNA replication (because there is no cytosine in the complementary sequence of CHH, mCHH is, per se established de novo). In contrast to DRM2-mediated methylation, the methyltransferases involved in these pathways act sequence specific: DNA METHYLTRANSFERASE 1 (MET1) complements missing methylation in the complementary strand of hemimethylated mCpG sites [7, 8], whereas non-CpG methylation(−maintenance) relies on the CHROMOMETHYLTRANSFERASES CMT3 (mCHG and to lesser extent mCHH) and CMT2 (mCHH and mCHG [107]). Chromomethyltransferases preferentially act on chromatin carrying H3K9me2. Histone methylation, in turn, is established by certain H3 lysine-9-specific methyltransferases of the Suvar3–9 subfamily (SUVH4 and its homologs SUVH5 and SUVH6) which are able to bind methylated DNA and preferentially act on histones in proximity of existing mCHG and mCHH [9, 15, 108–111].

Our RNA-seq data indicated contrasting transcriptional regulation of *SUVH6* - which was upregulated - and *CMT2* - of which one homolog (*Bv3_050080_yren*) was drastically downregulated in leaves of cold treated sugar beets (Fig. 8c). Under the assumption that sequence- or modification-specificities are conserved in sugar beet, an increase of SUVH6 should promote H3K9me2 at sites with pre-methylated CHG and CHH [110, 112]. H3K9me2, in turn, recruits CMT3 resulting primarily in mCHG maintenance. In the absence of CMT2, (possibly increased) H3K9me2 (placed by SUVH6) thus should favor mCHG through CMT3, whereas mCHH is expected to decrease. This is in line with the high ratio of dmCHH_hypo_:dmCHH_hyper_ and accordingly fits with a slightly higher number of hyper- compared to hypomethylated DMCs in CHG context. However, on a global scale, hypomethylation was observed in all cytosine contexts - including CpG, which indicates that reduction of methylation is not, or at least not exclusively due to reduced CMT-activity. Moreover, as shown in Fig. 7f, at least one of the two genotypes (GT1) we included in our analysis expressed relatively high levels of another close *CMT2* homolog, possibly substituting for the downregulation of *Bv3_050080_yren* in this genotype.

A considerable fraction of mCHH has been proposed to be dependent on CMT-, instead of RdDM-based DNA methylation. This mainly affects heterochromatin, where RdDM is blocked, whereas methylation via CMT2 can persist [11]. Accordingly, in case of severely reduced CMT2-activity, there should be an accumulation of dmCHH_hypo_ particularly in heterochromatic regions, which tend to be rich in TEs, but are usually characterized by a low density of protein coding genes. However, cold-dependent dmCHH was rather evenly distributed over chromosomes.

Considering the relatively high expression of another, possibly redundant CMT2-homolog in one of the genotypes in our study, the even distribution of differential CHH methylation across chromosomes, and rather ubiquitous hypomethylation (not only in CHH), we see no clear evidence for a major contribution of CMT2 to the observed effects of cold on methylation.

#### A ROS1-homolog might drive active DNA methylation in response to cold

In addition to passive loss of DNA methylation, plants utilize a complex enzyme machinery to actively erase DNA methylation at specific positions (Fig. 8d). Removal of cytosine-methylation in plants comprises excision of the entire methylated cytosine - as opposed to mere cleavage of the methyl-group - followed by repair of the cleavage site via insertion and ligation of an unmethylated cytosine [16–19]. Removal of the methylated cytosine in Arabidopsis is mediated by RELEASE OF SILENCING 1 (ROS1), DEMETER (DME) and DEMETER-LIKE 2 and 3 (DML2 and DML3), which are able to demethylate DNA irrespective of the sequence context of the modified cytosine [18, 113]. Following further modification of the cleavage site, the gap is (filled and) repaired via DNA polymerase and ligase enzymes, e.g. LIG1 in Arabidopsis [21]. Again, linking histone- to DNA modification, DME requires histone linker H1 and the histone remodeling complex FACT to demethylate DNA in heterochromatin during reproduction [114–116]. Similarly, the demethylase ROS1 is recruited to chromatin containing the histone variant H2A.Z. H2A.Z, in turn, is incorporated into histone octamers by the SWR1 complex, which is recruited to histone acetylation marks (via SWR1-associated MBD9 and NPX1) formerly added to the chromatin by the INCREASED DNA METHYLATION (IDM) complex [20, 117–119].

We found numerous sugar beet homologs putatively mediating DNA demethylation in the described pathways. Their transcriptional changes upon cold treatment collectively support that the overall loss of DNA methylation upon cold exposure was predominantly caused by an increase of active DNA demethylation (Fig. 8d): Cold triggered significant upregulation of *NPX1* expression, and also significantly enhanced expression of two SWR1 components, i.e. *PIE1* and *SWC4*, which overall might promote demethylation by ROS1 via deposition of H2A.Z. Finally, expression of *ROS1* itself was significantly upregulated upon cold treatment.

Expression of a homolog of the putative DNA glycosylase At3g47830 | DEMETER-like protein 2 (*DML2*) was significantly lowered by cold. However, while disruption of the corresponding gene in Arabidopsis resulted in reduced methylation at sites that were heavily methylated in the wild-type, cytosine residues that were unmethylated or weakly methylated in WT, in fact, showed an increase in DNA methylation [120].

In several plant species, *ROS1* expression decreases when RdDM is inhibited, for example through knock-out of a core RdDM-component [121, 122]. In contrast, despite simultaneous downregulation of several RdDM-genes including NRDP1, cold exposure resulted in upregulated *ROS1* expression in sugar beets. This suggests that, either - instead of being linked to a general decrease of de novo methylation, the observed transcriptional repression of these RdDM components might rather reflect altered RdDM (for example via shift of targeted loci); or the regulatory mechanism that represses *ROS1* in parallel to RdDM in other plants, is not conserved in sugar beets.

In fact, a short helitron TE in the *ROS1* promoter of Arabidopsis, the so-called methylstat or MEMS (methylation monitoring sequence), which acts as a sensor for DNA methylation and (in this rather rare example) activates *AtROS1* expression upon becoming methylated [121, 122], appears to be absent from the promoter of the sugar beet homolog. Besides, methylation of the cold induced *ROS1* homolog of sugar beet (or of its upstream region comprising the promoter) was not significantly altered by cold.

Whereas upstream regions were overlapped by about equally many hyper- and hypomethylated DMRs in CpG context, about three quarters of all DMRs in CHH context coinciding with upstream regions were hypomethylated (Fig. 3e). Hypomethylation was also clearly favored regarding DMCs occurring within 5′ flanks, − particularly regarding non-CpG positions, with about twice as many upstream DMCs in CHG, and more than six times as many cytosines in CHH context showing a significant reduction instead of an increase of methylation. The fact that we observed this particular association of hypomethylated DMCs with upstream regions, despite having a genome-wide larger number of hypermethylated DMCs in CHG, seems to imply that non-CpG hypomethylation is in some way specifically targeted towards those areas. As recently shown, ROS1 demethylates preferentially promoters of otherwise repressed genes [123]. As we detected a significantly upregulated expression of *ROS1* due to cold in our set of DEGs, we consider demethylation by ROS1 as a possible explanation for the observed association of hypomethylated (non-CpG) DMCs with 5′ regulatory regions. However, of the 33 genes carrying a hypomethylated DMC in non-CpG context in their upstream flanks, only one is also differentially expressed. This gene is a homolog of the flowering pathway gene *BBX32* from Arabidopsis, which in turn was shown to interact with CONSTANS-LIKE 3 (COL3) to target the promoter of *FT* [124], overall regulating the onset of flowering.

In contrast to RdDM-based methylation, CMTs show some specificity towards particular cytosine environments, i.e. they prefer to act on a cytosine directly preceding A and T rather than C [107]. Positions that were differentially methylated in response to cold treatment in our experiment revealed that hypermethylated CHG or CHH DMCs were almost completely devoid of another cytosine, particularly at the H directly following the cytosine that becomes (hyper)methylated (Additional File 1, Supplementary Fig. S4). In contrast, hypomethylated CHGs and CHHs were more tolerant towards additional cytosine residues. Based on Arabidopsis data, ROS1 has been proposed to counteract mainly RdDM to prevent spread of (TE-)methylation to genes [19, 125]. Under the assumption that hypomethylation is not primarily based on loss of RdDM, the increased frequency of CCG or CCH among hypo- compared to hypermethylated DMCs, fits with increased ROS1 activity counteracting methylation established through RdDM.

In summary, the transcriptional changes in sugar beet leaves in response to cold suggest an overall decrease of DNA methylation, mainly linked to enhanced active removal of methylated residues (mainly via ROS1).

## Conclusion

A plant’s ability to adapt gene expression to changes in the environment can be crucial to its survival and/or development and it is thought to be fine-tuned by DNA methylation. Our study provided insights into conserved methylomic and transcriptomic responses of sugar beets to cold exposure. We propose that cold-dependent reduction of DNA methylation was mainly due to active removal of methylation marks through collectively upregulated expression of sugar beet homologs within the ROS1 pathway. Mechanistically, these effects seem to be common among different plants including sugar beet, tea [126], tomato [65], and poplar [66]. Strikingly, while an overall reduced DNA methylation can theoretically be facilitated either via increase of demethylation, or through passive loss, cold appears to predominantly trigger upregulation of an active DNA demethylation pathway in all of these species.

## Methods

### Plant material and growth conditions

The sequencing data presented in this study was obtained from two hybrid sugar beet genotypes [4]. Seed material of *Beta vulgaris ssp. vulgaris* GT1 (KWS-accession: 1NB0218) and GT2 (KWS-accession: 1NB0133) used in this study were provided by, and cultivation and collection of material was performed at KWS SAAT SE & Co. KGaA (Einbeck, Germany). Plants were germinated and grown on standard soil substrate ED73 (Einheitserdwerke Patzer, Germany) mixed with sand (10% v/v) under short day conditions (10 h light/14 h dark), at 60% relative humidity and 110 μmol m^− 2^ s^− 1^ light intensity (fluorescent tube light). GT1 and GT2 beets were either grown under control conditions (20 °C during the day, 16 °C at night) for 15 weeks, or - after initial cultivation under control conditions for 12 weeks – were transferred to 12 °C for 7 days (acclimation phase), followed by 13 days at 4 °C and finally 24 h at 0 °C (cold treatment). Leaf material was collected from three biological replicates per genotype and condition, transferred to liquid nitrogen and stored at − 80 °C until further processing for methylomic and transcriptomic analysis.

### Sample preparation, sequencing and data availability

Sample extraction (total genomic DNA from leaves), library preparation and whole genome bisulfite sequencing (Illumina NovaSeq 6000 platform) was provided as a custom service (Novogene, Beijing, China). For WGBS, the PBAT (post-bisulfite adapter tagging, [127]) protocol for paired-end sequencing (PE150) was used. Total RNA for RNA-seq was extracted from leaf material as described in Martins Rodrigues et al. [4]. Poly-A selection, library preparation and stranded, paired-end sequencing (151 + 151; Illumina HiSeq 2000) was provided as a custom service (GATC GmbH, Konstanz, Germany). Raw sequencing data (RNA-seq and Whole Genome Bisulfite Sequencing) have been deposited to NCBI’s Sequence Read Archive under BioProject ID PRJNA74855, accessible via https://www.ncbi.nlm.nih.gov/sra/PRJNA748559 (also see paragraph Availability of Data and Materials in section Declarations). All software and parameters described in the following sections are additionally summarized in Additional File 3.

### Pre-processing and mapping

Raw sequence reads from both sequencing methods were inspected using fastQC [128] and multiQC [129]. Reads were then filtered for N-reads, common contaminants, and known adapter sequences from both ends of the reads using BBDuk (version 38.69, [130]); with k-mer length between 11 and 23). Reads were additionally filtered based on read quality (Q20) and length (minlen = 35 bp). WGBS reads had an additional step after adapter trimming in which the first 18 nucleotides of each read were removed (ftl = 18) using BBDuk (v38.69, [130]) as recommended by the company, to avoid bias due to the sequencing technique. Trimming success was confirmed based on quality reports generated for trimmed data using fastQC and multiQC again. Adapter- and quality-trimmed RNA-seq reads were mapped to RefBeet1.2.2 (RefBeet-1.2.fna.gz, [22]; downloaded from [50]), using STAR (v2.5.0a, [131]). Cleaned WGBS reads were mapped to the same reference genome using BISMARK (v22.3, [132]). Duplicated reads within the WGBS data were removed by BISMARK’s deduplication function.

### Methylation extraction and detection of DMCs and DMRs

The methylation status together with its positional coverage was evaluated based on BISMARK’s mapping alignments and methylation extractor function output (genome-wide cytosine report) for cytosines with a minimum read coverage of eight per position in each sample. Corresponding bam-files generated by BISMARK were used as input for detection of DMCs using the methylKit package (v1.19.0, [45]) within Bioconductor (v3.14, [133]) in R (v4.1.2 “Bird Hippie”, [134]), which was used to detect differentially methylated cytosines (comparisons of COLD vs CONTROL, for each cytosine context separately). A cytosine site with a (by default *SLIM* adjusted) *q-*value < .05 was defined as differentially methylated. Coverage-filtered BISMARK methylation extraction outputs were used for the detection of DMRs via HOME, a histogram-based machine learning approach (v1.0.0, [47]).

### Analysis of differential gene expression

Mapped transcriptomic reads were quantified on gene-level for all predicted protein coding genes (BeetSet-2.unfiltered_genes.1408.gff3, [44, 50]) using featureCounts v1.5.0. provided with the Subread package [135]. The output was used to analyze differential expression between CONTROL and COLD using DESeq2 (v1.39.0, [49]) with a reduced design formula, correcting for genotype specific differences in the transcriptomic cold response. Genes with an absolute Log_2_FoldChange ≥ 1 and an adjusted *p*-value (Bonferroni correction) of ≤ .05 were defined as differentially expressed (see Additional File 3 for main code snippets and non-default parameter settings).

### Pseudochromosome construction and visualization

For plots showing distribution of detected cold-responsive elements - i.e. of differentially expressed genes (DEGs), differentially methylated cytosines (DMCs), or differentially methylated regions (DMRs) - along chromosomes, all localized scaffolds (designated BvchrX.scaYYY), followed by all unplaced scaffolds (BvchrX_un.scaYYY, where X denotes the corresponding chromosome and YYY indicates the [incremental] number of the scaffold) assigned to the same chromosome were strung together with 10 kb pseudogaps inserted between individual sequence components (i.e. scaffolds). Based on the (cumulative) lengths of (preceding) scaffolds and pseudogaps, a position-adjustment table was generated, carrying - for each scaffold - a constant, from which relative positions of a given feature with respect to the corresponding (pseudo-)chromosome was calculated (based on the feature’s coordinates as given in the original annotation file, i.e. relative to its scaffold) directly prior to visualizing the data. If not otherwise specified, plots were generated using ggplot2 [136] in R [134] and finalized using Inkscape (v1.0.2–2, [137]).

### Functional classification and phylogenetic analysis

A fasta file containing protein sequences for all annotated BeetSet-2 genes [44] was obtained from ‘The *Beta vulgaris* Resource’ [50]. The file was checked for consistency (valid format, valid character usage etc.) using the Mercator4 Fasta Validator and sequences were assigned to MapMan bins using Mercator4 v3.0, both available from the ‘PlaBi dataBase’ [138]. Data for all 285 gene products assigned to MapMan category 12 (“Chromatin organization”) was extracted. Genes not assigned to any chromosome were discarded, leaving 267 genes. Of those, 38 were differentially expressed in response to cold. Following closer inspection of the DEGs assigned to MapMan category “Chromatin organization”, literature search on those genes (with a focus on those with predicted effects on cytosine modification) revealed 11 additional DEGs with homologs related to DNA methylation or demethylation. For the complete list of genes (MapMan-based as well as manually assigned) see Additional file 3.

Phylogenetic analysis was done based on amino acid sequences of DME/ROS- and chromomethyltransferase homologs from *Arabidopsis thaliana* and *Beta vulgaris*, as compiled in PLAZA HOM04D001046, or HOM04D001291, respectively [139]. Additionally, the peptide sequence of another putative nuclease that we found among our DEGs, and which in NCBI annotation data was described as “DEMETER-LIKE 2”, was included with the DME/ROS-set. Analyses were done based on these sequences for each set, independently, using default parameters of PhyML+SMS/OneClick-workflow available via NGphylogeny [140, 141], and subsequent visualization in iToL [142].

## Supplementary Information

**Additional file 1: Supplementary Figure S1.** Addition to Fig. 2: Methylation levels in the genome and in gene components under control conditions or after exposure to cold. **Supplementary Figure S2.** Addition to Fig. 2: Methylation levels of transposable and repetitive elements under control conditions or after exposure to cold. **Supplementary Figure S3.** Addition to Fig. 3: Overlaps of differential methylation with transposable elements. **Supplementary Figure S4.** Sequence composition in the neighborhood of fully methylated, non-methylated or differentially methylated cytosines. **Supplementary Figure S5.** Addition to Fig. 4: Methylation profiles along genes grouped by their expression level.

**Additional file 2: Supplementary Table S1.** Merged annotation and Arabidopsis homologs of all DEGs. **Supplementary Table S2.** Databases used for annotation of DEGs and assignment of Arabidopsis homologs.

**Additional file 3.** Tools and Implementation.

## Data Availability

*Beta vulgaris subsp. vulgaris* lines used in this study can be obtained from KWS SAAT SE & Co. KGaA for non-commercial research purposes. Raw sequencing data (RNA-seq and Whole Genome Bisulfite Sequencing) have been deposited to NCBI’s Sequence Read Archive (submission ID: SUB9992759) under BioProject ID PRJNA748559, available via https://www.ncbi.nlm.nih.gov/bioproject/PRJNA748559. Refbeet1.2.2 [22] and corresponding annotations (BeetSet-2, [44]) were downloaded from ‘The *Beta vulgaris* Resource’ [50] and served as reference genome during mapping and any downstream analyses.
