# Supplementary material for: Multi-omics data integration reveals link between epigenetic modifications and gene expression in sugar beet (Beta vulgaris subsp. vulgaris) in response to cold
Source: BMC Genomics. 2022 Feb 17;23:144. doi: 10.1186/s12864-022-08312-2 (PMC8855596; doi:10.1186/s12864-022-08312-2)
Supplement: Supplementary file 1 — Additional file 1: Supplementary Figure S1. Addition to Fig. 2: Methylation levels in the genome and in gene components under control conditions or after exposure to cold. Supplementary Figure S2. Addition to Fig. 2: Methylation levels of transposable and repetitive elements under control conditions or after exposure to cold. Supplementary Figure S3. Addition to Fig. 3: Overlaps of differential methylation with transposable elements. Supplementary Figure S4. Sequence composition in the neighborhood of fully methylated, non-methylated or differentially methylated cytosines. Supplementary Figure S5. Addition to Fig. 4: Methylation profiles along genes grouped by their expression level. [file 12864_2022_8312_MOESM1_ESM.pdf]

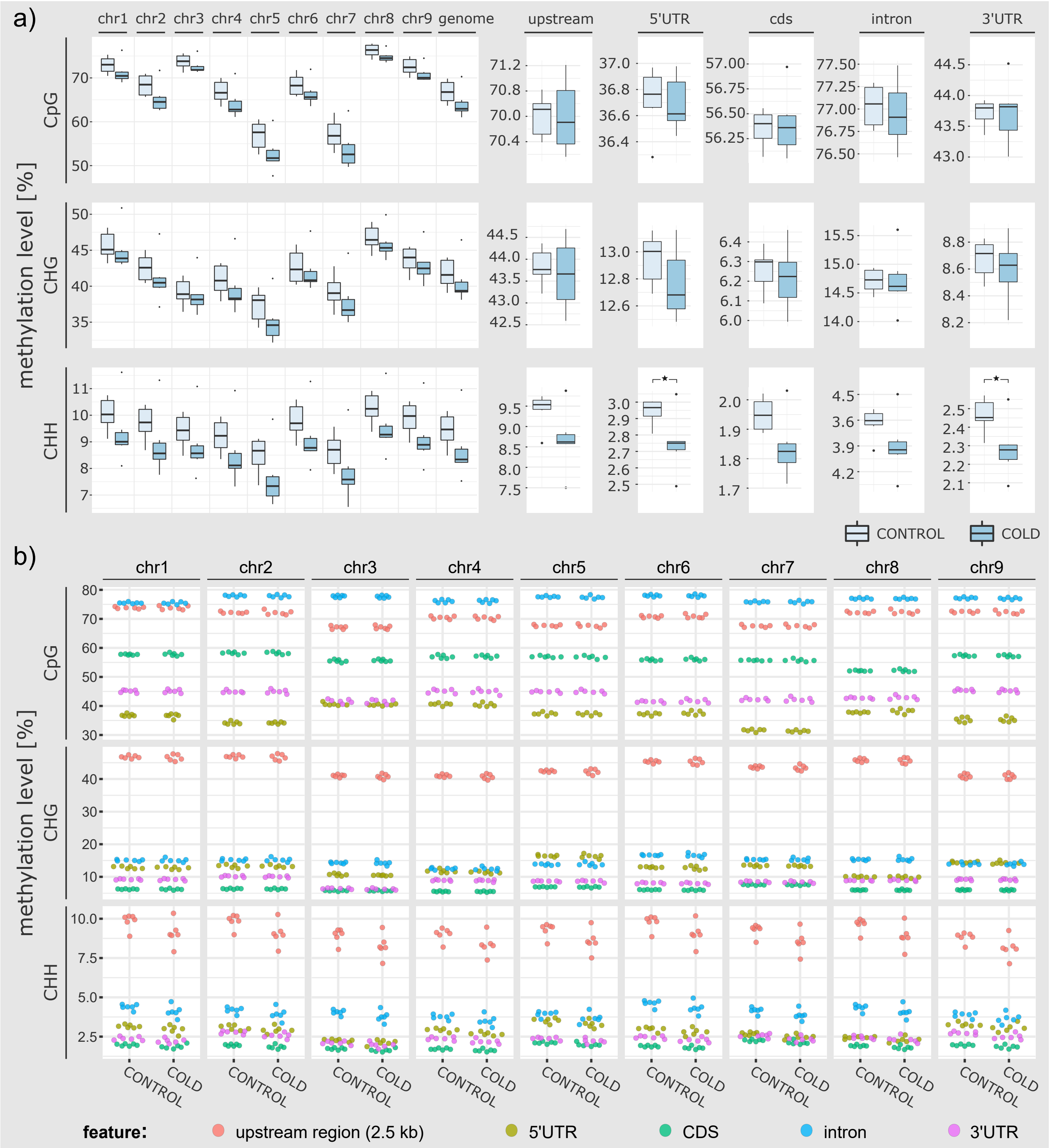

Supplementary Figure S 1. Addition to Figure 2: Methylation levels in the genome and in gene components under control conditions or after exposure to cold. a) Global methylation levels of individual chromosomes, the entire genome (panels on the left), or within gene components (panels on the right) with light and dark blue boxes representing methylation levels of CONTROL and COLD samples, respectively. Asterisks indicate significant differences (p-value $\leq$ .05; one-sided t-test) between methylation levels of CONTROL and COLD. Note that the plots shown panel a) are based on the same data as those depicted in Figure 2 (main article), but with y-axes scaled to fit data of individual subplots. b) Methylation levels of gene components and upstream regions (comprising 2.5 kb 5' of TSSs) plotted per chromosome. Per sample methylation levels were extracted for the total of cytosines of an individual feature (e.g. all cytosines within the introns of a particular gene). For a given feature and chromosome, weighted averages were then determined (on sample-level) by taking into account the number of cytosines that had been assigned to an individual feature, initially (for a particular feature of an individual gene this number is the same for different samples). Dots represent the weighted average for individual samples in each group (CONTROL & COLD).

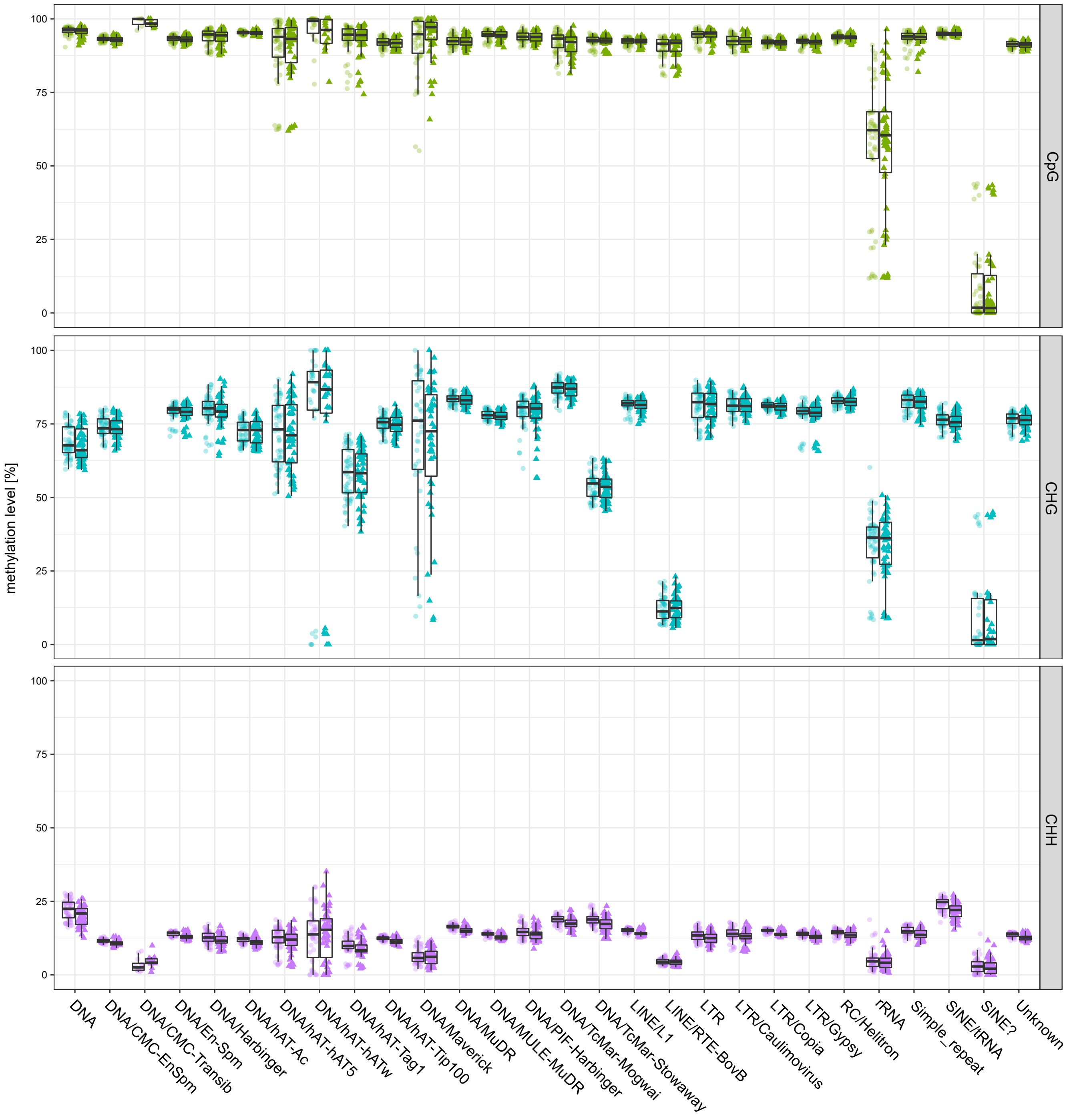

Supplementary Figure S 2. **Addition to Figure 2. Methylation levels of transposable and repetitive elements under control conditions or after exposure to cold.** Methylation levels of different transposable element families with lighter and darker shades representing methylation levels of TEs in CONTROL and COLD samples, respectively. Methylation of cytosines is shown separately for each sequence context (mCpG, mCHG, mCHH). Individual datapoints represent TE family methylation levels of an individual sample averaged per chromosome and the depicted repeat family.

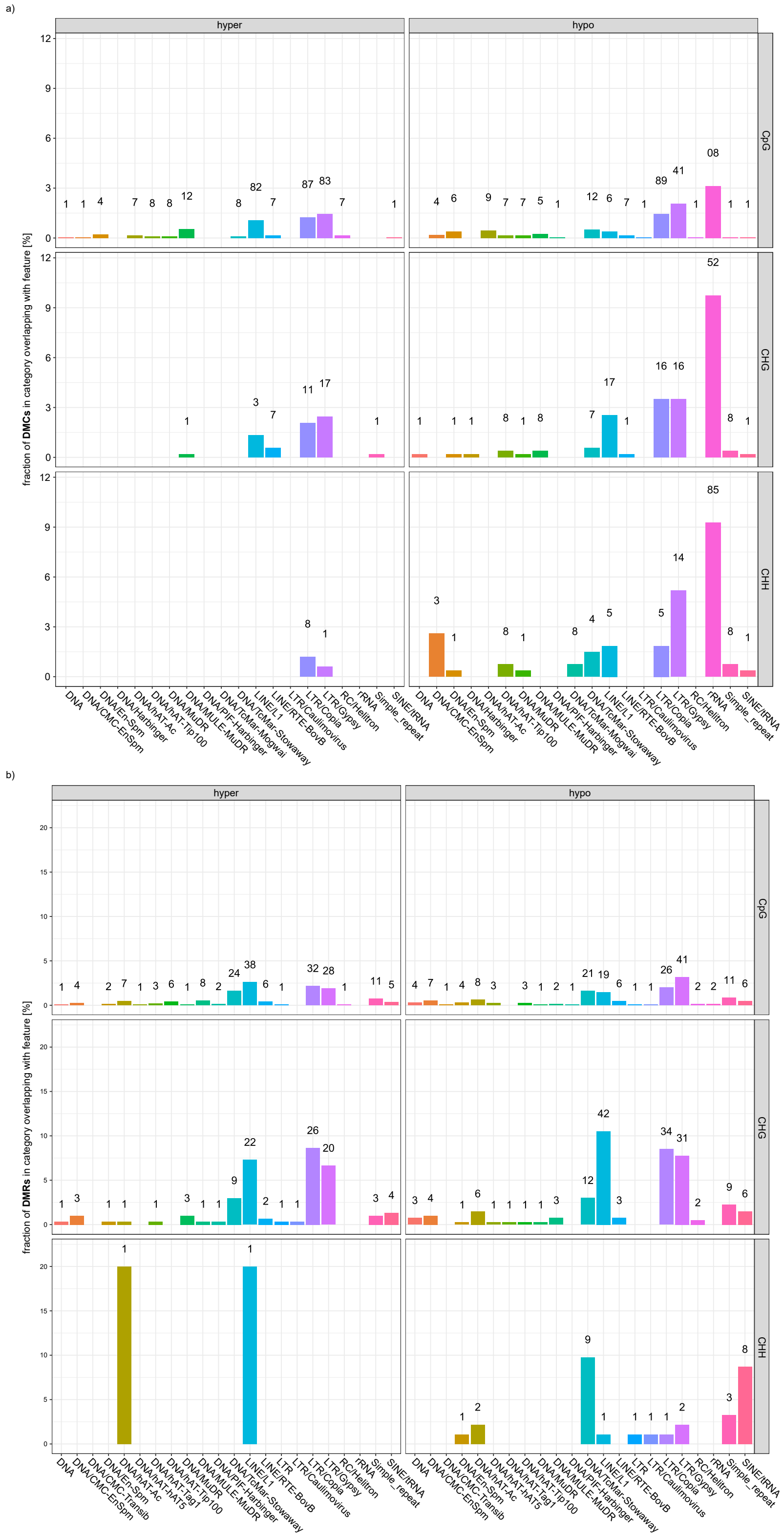

Supplementary Figure S 3. **Addition to Figure 3: Overlaps of differential methylation with transposable elements.** Fraction of hyper-methylated (left) or hypomethylated (right) a) DMCs, or b) DMRs in CpG, CHG or CHH context overlapping a transposable element (TE) of the respective family. Percentages (x- axis) refer to the fraction of associated DMCs (in a) (or DMRs, in b) compared to all DMCs (or DMRs) of the same context and the same direction of change in methylation, respectively. Numbers indicate the count of differentially methylated positions or regions constituting the corresponding bar.

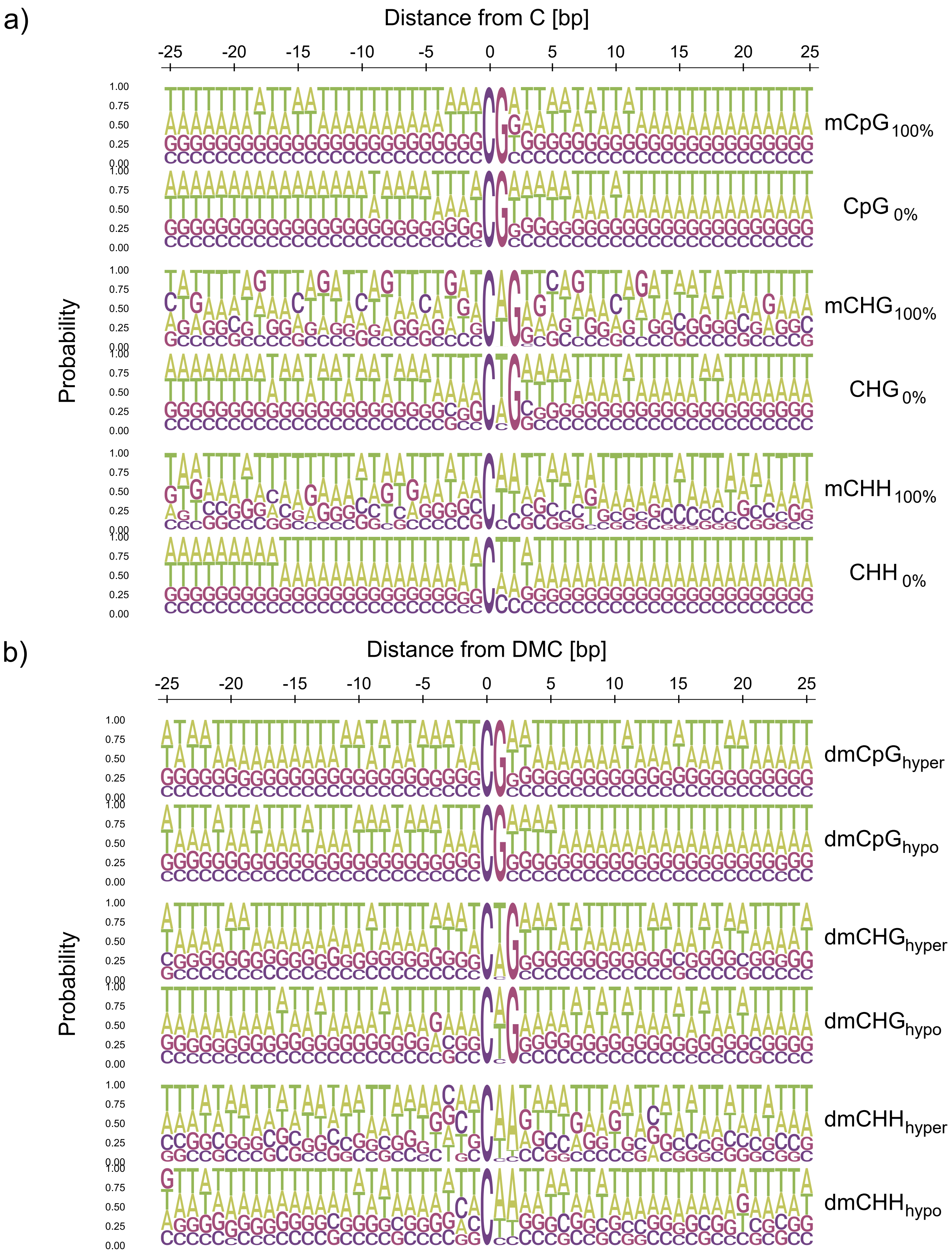

Supplementary Figure S 4. **Sequence composition in the neighbourhood of fully methylated, non-methylated or differentially methylated cytosines.** a) & b) Sequence logos depicting nucleotide frequencies along 25 bp-windows up- and downstream of a) consistently methylated ($mCXX_{100\%}$), or of consistently non-methylated ($CXX_{0\%}$ cytosines, and of b) cytosines differentially methylated (dm) between COLD and CONTROL. Labels on the right designate DMCs with significantly lower ($dmCXX_{hypo}$) or higher ($dmCXX_{hyper}$) methylation levels detected in COLD compared to CONTROL.

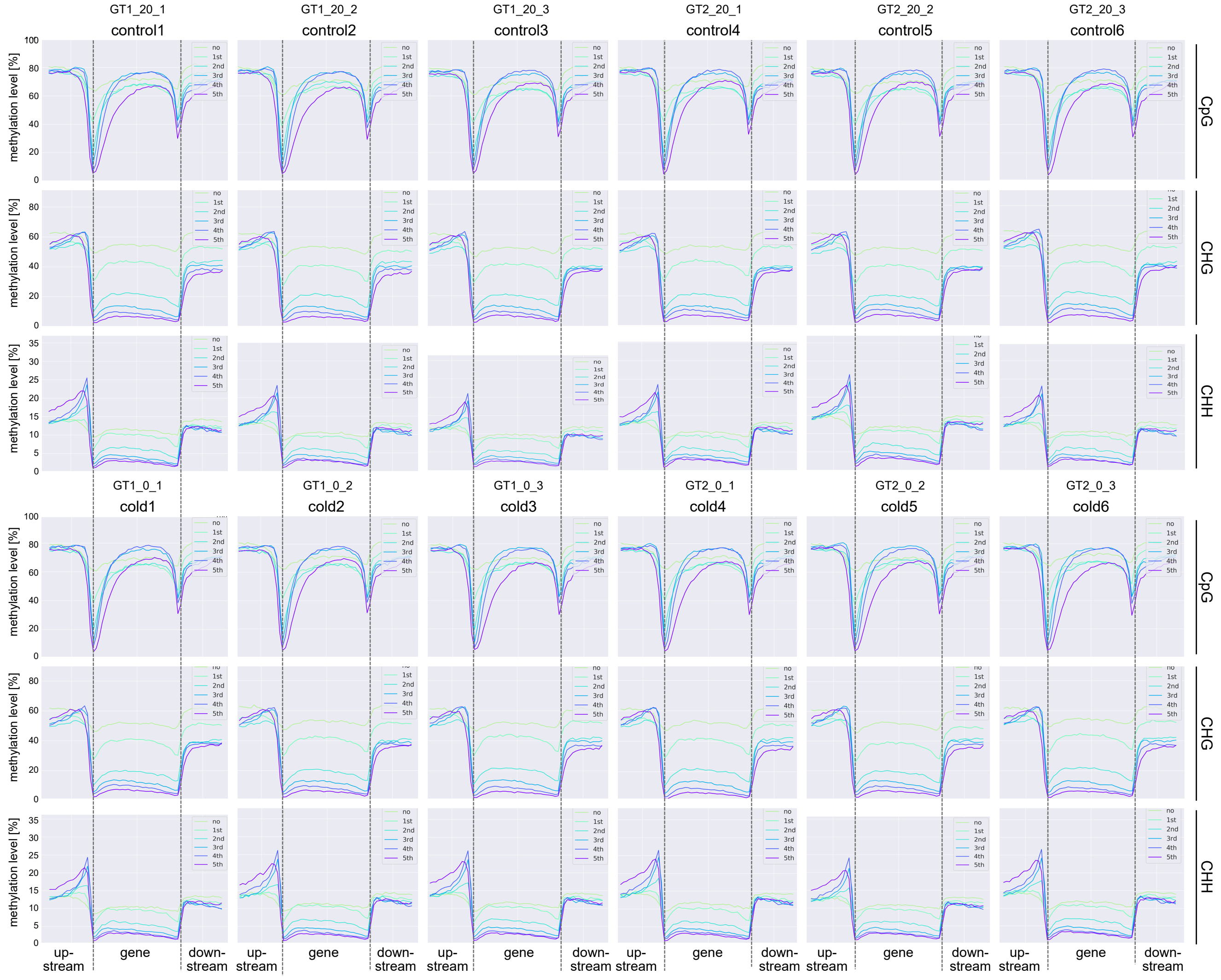

Supplementary Figure S 5. **Addition to Figure 4: Methylation profiles along genes grouped by their expression level.** Each (sub-)plot depicts the distribution of cytosine methylation along all genes and their flanking regions in DNA of individual sugar beets grown under control conditions or after exposure to cold. Each plot depicts data corresponding to cytosines in the indicated (right side) context of one individual sample. For each sample, genes were ranked and assigned to one of six groups based on their expression level (FPKM; i.e. after normalizing for gene lengths). Each gene was split into 30 windows (TSS to TTS, including introns) and the distribution of methylation along all genes with similar expression (in that sample) is averaged and shown as an individual line. Different colors thus indicate methylation of genes with different expression levels, with lighter colours representing methylation levels along silent or lowly expressed genes, and darker colors representing methylation along highly expressed genes for a given cytosine context. Each flank extends another 15 windows (half the gene length) 5' of the TSS (upstream) or 3' of the TTS (downstream), respectively.
