# Supplementary material for: Multi-omics data integration reveals link between epigenetic modifications and gene expression in sugar beet (Beta vulgaris subsp. vulgaris) in response to cold
Source: BMC Genomics. 2022 Feb 17;23:144. doi: 10.1186/s12864-022-08312-2 (PMC8855596; doi:10.1186/s12864-022-08312-2)
Supplement: Supplementary file 3 — Additional file 3. Tools and Implementation. [file 12864_2022_8312_MOESM3_ESM.pdf]

Additional File 3

TOOLS AND IMPLEMENTATION

All used commands are listed below and are separated for the RNA sequenced samples (RNAseq analysis) and the whole genome bisulfite sequenced samples (WGBS analysis). If not explicitly mentioned, all software specific parameters were used by default. If specific packages are used within a used program, it is listed additionally in the relevant section.

RNAseq analysis

I. QUALITY CHECK

FASTQC, Version: 0.11.8

```
fastqc /path/to/fastqcfile.fq.gz
```

MULTIQC, Version: 1.7

```
multiqc /path/to/fastqcfiles
```

II. TRIMMING

BBDuk, Version: 38.69

```
bbduk.sh in1=/path/to/inputfile1.fq.gz
in2=/path/to/inputfile2.fq.gz
out1=/path/to/outputfile1.fq.gz
out2=/path/to/outputfile2.fq.gz
outs=/path/to/outputSingletons.fq.gz
ref=/path/to/adapters.fa ktrim=rl k=23 mink=11 hdist=1
tbo tpe qtrim=rl trimq=20 minlen=35
```

III. QUALITY CHECK 2

FASTQC, Version: 0.11.8

```
fastqc /path/to/.../fastqcfile.fq.gz
```

MULTIQC, Version: 1.7

```
multiqc /path/to/.../fastqcFiles
```

IV. MAPPING

STAR, Version: 2.5.0a

```
STAR --runThreadN 12 --runMode genomeGenerate --genomeDir
/path/to/genomeIndices --genomeFastaFiles
/path/to/referenceGenome.fna –sjdbGTFfile
/path/to/annotationFile.gff3 --sjdbOverhang 149 --
sjdbGTFfeatureExon CDS --sjdbGTFtagExonParentTranscript
ID --sjdbGTFtagExonParentGene Parent
```

```
STAR --runThreadN 12 --readFilesCommand zcat --
readFilesIn /path/to/inputfile1.fq.gz
/path/to/inputfile2.fq.gz --genomeDir
/path/to/genomeIndices --alignIntronMax 1000 --outSAMtype
BAM SortedByCoordinate --outReadsUnmapped Fastx --
outFileNamePrefix OutputFilePrefix
```

```
STAR --runThreadN 12 --readFilesCommand zcat --
readFilesIn /path/to/inputfile1.fq.gz
/path/to/inputfile2.fq.gz --genomeDir
/path/to/genomeIndices --alignIntronMax 1000 --
sjdbFileChrStartEnd /path/to/FileSJ.out.tab --outSAMtype
BAM SortedByCoordinate --outReadsUnmapped Fastx --
outFileNamePrefix OutputFilePrefix
```

V. FEATURE COUNTING

FEATURECOUNTS, Version: 1.5.0

```
featureCounts -p -M --primary -t gene -g Name -s 0 -G
/path/to/RefBeet-1.2.fna -a path/to/annotationFile.gff3
-T 10 -o /path/to/OutputFilename.txt
/path/to/inputfile1.bam /path/to/inputfile2.bam …
/path/to/inputfileN.bam
```

VI. DIFFERENTIAL GENE EXPRESSION ANALYSIS

R, Version: 4.0.2 - 4.1.2 “Bird Hippie”
DESEQ2, Version: 1.39.0

```
# design: control for genotype-dependent cold-response wile testing effect of temperature


dds <- DESeq2::DESeqDataSetFromMatrix(countmatrix, colData = sampledata, rowRanges = rowdata, design = ~ genotype + temp + genotype:temp)


dds <- estimateSizeFactors(dds)


fpkms <- fpkm(dds)

normcounts <- counts(dds, normalized = TRUE)


res <- DESeq2::results(dds4, alpha = 0.05, pAdjustMethod = "bonferroni")


reswald.05bonf <- DESeq2::results(dds, name="temp_C0_vs_C20",
                                  test="Wald",
                                  alpha = 0.05,
                            pAdjustMethod = "bonferroni")
```

WGBS analysis

I. QUALITY CHECK

FASTQC, Version: 0.11.8

```
fastqc /path/to/file.fq.gz
```

MULTIQC, Version: 1.7

```
multiqc /path/to/fastqcFiles
```

II. TRIMMING

BBDuk, Version: 38.69

```
bbduk.sh in1=/path/to/inputfile1.fq.gz
in2=/path/to/inputfile2.fq.gz
out1=/path/to/outputfile1.fq.gz
out2=/path/to/outputfile2.fq.gz
outs=/path/to/outputSingletons.fq.gz
```

```
ref=/path/to/adapters.fa ktrim=rl k=23 mink=11 hdist=1
tbo tpe qtrim=rl trimq=20 ftl=18 minlen=35
```

III. QUALITY CHECK 2

FASTQC, Version: 0.11.8

```
fastqc /path/to/file.fq.gz
```

MULTIQC, Version: 1.7

```
multiqc /path/to/fastqcFiles
```

IV. MAPPING

BISMARK, Version: 22.3

```
bismark_genome_preparation --parallel 6 --verbose
/path/to/genome_preparation_file

bismark --non_bs_mm --output_dir /path/to/ mapping_Output
--unmapped --genome_folder
/path/to/folder/containing/ReferenceGenomeAndAnnotation
/path/to/inputfile1.fastq.gz /path/to/inputfile2.fastq.gz
… /path/to/inputfileN.fastq.gz

deduplicate_bismark --paired --output_dir
/path/to/deduplication_Output --bam
/path/to/inputfile1.bam /path/to/inputfile2.bam …
/path/to/InputFileN.bam

bismark_methylation_extractor --paired-end --scaffolds --
bedGraph --cytosine_report --CX_context --
split_by_chromosome --cutoff 8 -o
/path/to/methylationExtractionOutput --genome_folder
/path/to/ReferenceGenomeAndAnnotation
/path/to/inputfile1.bam /path/to/inputfile2.bam ...
/path/to/inputfileN.bam
```

V. METHYLATION PROFILING ALONG GENES

HOME, Version: 1.0.0

```
python MethGET/preprocess.py -s SampleList.txt -g
GeneAnnotationFile.gtf
```

```
python MethGET/metagene.py -n SampleName -p region -
No_bins 30
```

VI. DMC DETECTION

R, Version: 4.1.2
methylKit, Version: 1.19.0

```
sample.ids <- list("GT1_20_1", "GT1_20_2", "GT1_20_3",
                   "GT2_20_1", "GT2_20_2", "GT2_20_3",
                   "GT1_0_1", "GT1_0_2", "GT1_0_3",
                   "GT2_0_1", "GT2_0_2", "GT2_0_3")

treatment <- c(1,1,1,1,1,1,0,0,0,0,0,0)

# reads and processes sorted BAM files (one per sample) and stores processe
d data as tabix files (one per context and sample)
db.raw.list <- processBismarkAln(location=as.list(bis.BAM.files),
                             sample.id = sample.ids,
                             assembly = "Refbeet1.2.2",
                             save.folder="./input",
                             save.context = c("CpG", "CHG", "CHH"),
                             read.context = "none",
                             # for paired-end:
                             nolap = TRUE,
                             mincov = 8,
                             minqual = 20,
                             phred64 = FALSE,
                             treatment = c(1,1,1,1,1,1,0,0,0,0,0,0),
                             save.db = TRUE)
```

```
# saved tabix files (.bgz; for individual samples) can be read via:
```

```
CpG.files <- list.files("./input/methylDB.CpG", "^(GT1|GT2).*\\.bgz$", full
.names = TRUE)
```

```
# db.raw.list.CpG can be created from single databases created via processB
ismarkAlign:
db.raw.list.CpG <- methRead(as.list(CpG.files),
                       treatment=c(1,1,1,0,0,0,1,1,1,0,0,0),
                       dbtype = "tabix",
                       save.db = TRUE,
                       dbdir = "methylDB.CpG",
                       mincov = 8)
```

```
# To create one object for all samples ("baseDB"):
db.base.CpG <- methylKit::unite(db.raw.list.CpG, destrand = FALSE)
```

```
# or, when a base.db object has already been created:
base.CpG.file <- "./methylDB.CpG/methylBase_CpG.txt.bgz"
base.CpG <- readMethylDB(base.CpG.file)
```

```
# creates a matrix containing percent methylation values
perc.meth.CHH <- percMethylation(base.CHH, rowids = TRUE)
```

```
# DMC detection:
dmc.CpG <- calculateDiffMeth(base.CpG,
                             dbdir = "./input/methylDB.CpG",
                             suffix = "DMC-CpG",
                             dbtype = "tabix",
                             save.db = TRUE,
                             mincov = 8)
```

```
dmc.CpG.05 <- getMethylDiff(dmc.CpG,
                            qvalue=0.05,
                            difference = 0,
                            suffix = "q05")
```

VII. DMR DETECTION

HOME, Version: 1.0.0

```
HOME-pairwise -i /path/to/samplefile/samplefile.txt -t CG -wrt C20 -
npp 10 -o /path/to/output/directory/CpG &&
```

```
HOME-pairwise -i /path/to/samplefile/samplefile.txt -t CHG -wrt C20 -
npp 10 -o /path/to/output/directory/CHG &&
```

```
HOME-pairwise -i /path/to/samplefile/samplefile.txt -t CHH -wrt C20 -
npp 10 -o /path/to/output/directory/CHH
```
